# Promising bioactive metabolites of mangrove inhabitant *Streptomyces tauricus* and prostate cancer PC3 cell inhibition by antimicrobial peptides

**DOI:** 10.3389/fmicb.2023.1152985

**Published:** 2023-06-16

**Authors:** Yalpi Karthik, Manjula Ishwara Kalyani, Srinivasa Krishnappa, Krishnaveni Ramakrishna, Samy M. Sayed, Ohud Muslat Aharthy, Seham Sater Alhelaify, Muntazir Mushtaq

**Affiliations:** ^1^Department of Studies and Research in Microbiology, Mangalore University, Kodagu, Karnataka, India; ^2^Department of Studies and Research in Biochemistry, Mangalore University, Kodagu, Karnataka, India; ^3^Department of Studies and Research in Microbiology, Vijayanagara Sri Krishnadevaraya University, Ballari, Karnataka, India; ^4^Department of Science and Technology, University College-Ranyah, Taif University, Taif, Saudi Arabia; ^5^Department of Biotechnology, Faculty of Science, Taif University, Taif, Saudi Arabia; ^6^MS Swaminathan School of Agriculture, Shoolini University of Biotechnology and Management Sciences, Solan, Himachal Pradesh, India

**Keywords:** anticancer, chromatography, FESEM, mangroves, *Streptomyces tauricus*

## Abstract

*Streptomyces* is a group of microbes known for antibiotic production and has contributed to more than 70% of present commercially available antibiotics. These antibiotics are important in the management, protection, and treatment of chronic illnesses. In the present study, the isolated *S. tauricus* strain from mangrove soil in Mangalore, India (GenBank accession number: MW785875) was subjected for differential cultural characterization, phenotype involving brown pigmentation, filamentous mycelia, and ash-colored spore production was observed using field emission scanning electron microscopy (FESEM) analysis revealing filamentous mycelia possessing a straight spore chain. Spores were visualized as elongated, rod-shaped, smooth surfaces with curved edges. After optimized growth conditions for *S. tauricus* on starch-casein agar medium, the GC/MS analysis of *S. tauricus* intracellular extract detected bioactive compounds reported for pharmacological applications. Analyzed using the NIST library, most of the bioactive compounds identified in intracellular extract had molecular weights of less than 1 kDa. On the PC3 cell line, the Sephadex G-10 partially purified eluted peak protein fraction demonstrated significant anticancer activity. The LCMS analysis revealed the presence of Tryprostatin B, Fumonisin B1, Microcystin LR, and Surfactin C with molecular weights below 1 kDa. This study found that small molecular weight microbial compounds are more effective in a variety of biological applications.

## Introduction

Mangrove habitat possesses unique environmental conditions characterized by high tidal forces, hypersaline conditions, substantial changes in temperature, and ideal flora and fauna diversity (Karthik et al., 2020). These vulnerable conditions are helpful for microbes in adjusting themselves to any extreme environment. This habitat will serve as a prominent repository for the isolation of biologically important compounds (Alongi, 2015). *Actinomyces are Gram-positive filamentous bacteria that differ in color and produce spores when mature* (Chater, 2013), and share the characteristics of bacteria and fungi. The genetic and environmental adaptability of the *Actinomyces* group supports the production of valuable and biologically important compounds. Among the *Actinomyces,* the genus *Streptomyces* shares more than 70 percent of the active compounds with the pharmaceutical industry for the treatment of various ailments (Chater, 2013; Karthik et al., 2023).

These *Streptomyces* are broadly used in the control, prevention, and treatment of diseases through the production of bioactive compounds. They are widely used as antibiotics, anti-cancer, anti-tumor, anthelmintic, anti-oxidant, anti-diabetic, immunosuppressors, neuritogenic, anti-inflammatory, anti-angiogenic, anti-algal, anti-fungal, anti-parasitic, anti-malarial, anti-viral, anti-bacterial and many more biological applications (Nishioka and Katayama, 1978; Renner et al., 1999; Fernebro, 2011; Janardhan et al., 2014; Desouky et al., 2015; Abd-Elnaby et al., 2016).

The *S. lividans* is involved in post-translational modification by acylation at lysine residue of active site for production of CoA ligases and many organic acids (Burckhardt et al., 2020). The cyclic antimicrobial peptide receptor protein and novel lipopeptide daptomycin are produced by *S. roseosporus* (Wu et al., 2021). These studies made us to search alternative sources of antimicrobial peptides from different mangrove sources. One such prominent strain was described in detail in our previous study on isolation procedure as well as the microscopic and macroscopic characters identified as *S. tauricus* with the GenBank accession number MW785875 (Karthik and Kalyani, 2022). The intracellular protein, extraction, estimation, and purification, along with their potential antimicrobial activity, were observed against test pathogens, *S. typhimurium, S. aureus, P. aeruginosa, B. cereus,* and *P. vulgaris* cultures. The protein was characterized through LCMS and SDS PAGE techniques, and small peptides were detected (Karthik and Kalyani, 2022).

In the present study, the optimization of suitable growth media for *S. tauricus* and its micromorphology were analyzed using FESEM (field emission scanning electron microscopy). The isolation of an intracellular extract of *S. tauricus* was characterized through GCMS and LCMS. This GC-MS analysis showed the maximum number of small bioactive compounds having efficient biological activities, whereas the LCMS characterization detected Tryprostatin B, FumonisinB1, Microcystin LR, and Surfactin C having a low molecular weight and exhibiting an effective anticancer activity against a prostate cancer cell line compared with standard cisplatin.

## Materials and methods

### Mangrove soil collection

The soil sample was collected from mangroves in the Dakshina Kannada region of Mangalore. The Ashok Nagar, Tannira Bavi (ANTB) sample collection site is located at 12°54′16.5″N and 74°49′09.4″E. At the site of the collection, the soil had a sandy texture and was black, and the environmental factors of pH 7.2 and temperature of 21°C were documented. In sterile containers, the samples were taken to the Molecular Research Laboratory (MRL), Division of Microbiology, Jnana Kaveri, Mangalore University, India. The soil samples were pre-heated for 2 h at 60°C before serial dilution and isolation to prevent bacterial and fungal growth (Mohan et al., 2013; Sridevi et al., 2015; Sapkota et al., 2020).

### Isolation and culture characteristics of mangrove soil actinomyces

One gram of soil sample was serially diluted seven folds and aliquots of 100 μL of each dilution were spread out on SCN plates in triplicates (starch: 10 g, casein: 0.30 g, KNO3: 2 g, MgSO4.7H2O: 0.050 g, K2HPO4: 2 g, NaCl: 2 g, CaCO3: 0.20 g, FeSO4: 0.010 g in 1 L of dH2O, pH 7.2) and incubated at 30°C ± 2°C for 7 days. The plates were screened for Actinomyces colonies and single colonies were selected, sub-cultured on SCN media (Karthik et al., 2020; Wang et al., 2020). The culture characteristics were visualized and recorded such as color of aerial and substrate mycelia, and diffusible pigments production.

### Cultural characteristics

The isolated *S. tauricus* strain was subjected to FESEM analysis at different objective distances, spore structure (1 and 2 μm) mycelial structure (10 and 20 μm) to visualize the complete micromorphological structures. The sequencing and identification of *S. tauricus* were reported in our previous work (Karthik and Kalyani, 2022).

### Optimization of growth media for *Streptomyces tauricus*

The five distinct media were used to analyze the phenotypic characteristics of potential *Actinomyces* isolates. The media that were used are malt extract agar (MEA) malt extract: 30.0 g, peptone (5.0 g), and agar (15 g). Sucrose peptone agar (SPA) was prepared using sucrose (20.0 g), peptone (5.0 g), K_2_HPO_4_ (0.50 g), MgSO_4_.7H_2_O (0.25 g), and agar 15.0 g. The glucose asparagine agar (GAA) was prepared using glucose (10 g), asparagine (0.50 g), K_2_HPO_4_ (0.50 g), and agar (15.0 g). Nutrient agar (NA) was prepared using peptone: 5.0 g, sodium chloride: 5.0 g beef extract: 3.0 g. The yeast extract agar (YEA) was prepared using yeast extract (3.0 g), peptone (5.0 g), and agar (15.0 g). These different media were used for growth studies of potential *Actinomyces* isolates.

### Intracellular extract

*Streptomyces tauricus* was grown in SCN broth for 7–10 days at 30°C ± 2°C with constant agitation at 100 rpm. The separated cultivated biomass cells were centrifuged at 7,000 rpm for 8 min, twice washed in phosphate-buffered saline devoid of Mg^2+^ and Ca^2+^, and then centrifuged one more time. The cells were then suspended in 10 mL of ice-cold acetone, allowed to stand on ice for 5 min, then centrifuged for 8 min at 7,000 rpm. Following the removal of any remaining acetone with a stream of nitrogen, the intracellular extract was incubated with 1.0 mL of 1% SDS for 2 min. Spectrometric (LC-MS and GC-MS) methods were used to characterize this intracellular extract, and it was analyzed using NIST library.

### Gas chromatography-mass spectroscopic analysis

The GC–MS studies of the intracellular extract of *S. tauricus* were performed with a Perkin Elmer Gas Chromatograph Clarus 680 and a Perkin Elmer Mass Spectrometer Clarus SQ 8C. The equipment has a Perkin Elmer Elite-5MS standard column with dimensions of 30 m long × 0.250 mm inner diameter × 1 micron (60°C–350°C). With a split ratio of 10:1, the injected volume of 2 μL was completely run for 26.6 min. The carrier gas used is helium, with a flow rate of 2 mL/min. The source temperature was operated at 230°C, and the inlet temperature was operated at 250°C. The oven temperature was initially set to 80°C with a 2.0 min hold time; ramp 1 was 10.0/min to 150°C with a 1.0 min hold time; and ramp 2 was 15.0/min to 250°C with a 10.0 min hold time. Comparing them to those in the NIST computer library allowed the components to be identified, which were connected to the GC-MS apparatus, and the findings were reported.

### Liquid chromatography-mass spectrophotometer

The peak fraction obtained from Sephadex G-10 was analyzed and subjected to LC-MS; Model Synapt G2 a method of analytical chemistry was used that combine liquid chromatography’s physical separation capabilities with other methods. With mobile phase A: 0.1% Formic acid in water and mobile phase B: 0.1% formic acid acetonitrile with the mass analysis capabilities of mass spectrometry (MS) a vacuum degasser, binary pump, and well-plate auto sampler were included in an Agilent 1,100 LC system. The BEH C18, 50 mm × 1.0 mm, 1.7 μm C18 column was used (Waters United States) compared to the NIST computer library.

### MTT assay

The *in vitro* studies were conducted using prostate cancer cells that were grown in T-25 flasks. Trypsinized PC3 cells were aspirated into a 5 mL centrifuge tube, which were centrifuged for 300 rpm and the cell pellet were separated. The cell count was adjusted in DMEM-HG media to produce a 200 μL suspension with approximately 10,000 cells. Using an ESCO model CLM170B-8-UV CO_2_ incubator, 200 μL of the cell suspension was put into each well of the 96-well microtiter plate. The plate was then incubated at 37°C with 5% CO_2_ for 24 h. The used medium was aspirated 24 h later. The standard medication cisplatin and several test drug concentrations totaling 200 μL each was introduced to the corresponding wells. The plates were then incubated for 24 h at 37 degrees Celsius with 5% CO_2_. The drug-containing medium was aspirated when the plate was taken out of the incubator. The plate was incubated at 37°C with 5% CO_2_ for 3 h after 200 μL of media containing 10% and 0.5 mg/mL of MTT reagent were added to each well. Without disrupting the crystals that had grown, the culture medium was removed. The produced formazan was then solubilized by adding 100 μL of solubilization solution (DMSO) and gently shaking the plate in a gyrator shaker. The absorbance was measured using a microplate reader of the Multiskan sky ELISA spectrophotometer at a wavelength of 570 nm and also at 630 nm (Karthik et al., 2023).

## Results and discussion

The Ashok Nagar, Tannir Baavi mangrove region in Mangalore, India, served as a suitable source for isolating 20 distinct *Actinomyces* cultures. Among these 20 distinct *Actinomyces* cultures, the *S. tauricus* (GenBank accession number MW785875) was isolated, and their biological activities were reported in our previous work (Karthik and Kalyani, 2022). In continuation to previous work, the *S. tauricus* strain was initially examined for morphological characteristics after FESEM analysis. GCMS was also used to characterize biologically important chemical components found in the intracellular extract of the *S. tauricus* strain, and LCMS was used to characterize a partially purified protein sample, which revealed a significant number of bioactive compounds.

On starch casein nitrate agar medium, the cultural characteristics of the mangrove-adapted *S. tauricus* strain showed white filamentous mycelia and at maturity, ash-colored spores. Brown pigmentation was observed to be produced on SCNA media. Gram staining was found to be positive after further microscopic examination.

**Figure 1:**
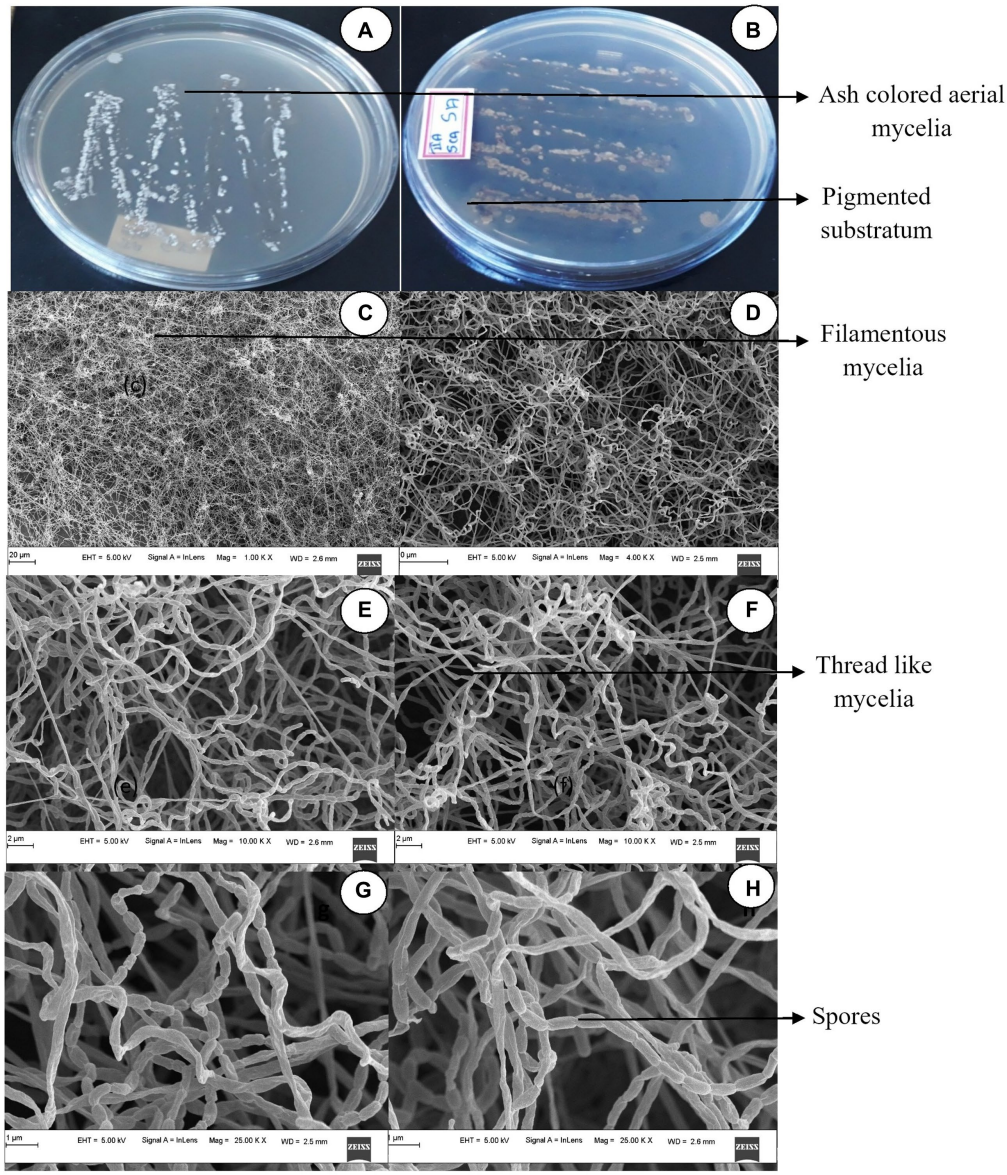
Cultural characteristics of *Streptomyces tauricus*. **(A)** Front view. **(B)** Rear view. **(C,D)** Mycelia observations under FESEM. **(E,F)** Mycelia along with spore analysis under FESEM **(G,H)** Spore structure analysis using FESEM.

The isolate when further subjected to FESEM microscopic studies revealed the mycelial morphological characteristics of the genus *Streptomyces.* Further, filamentous mycelia possessed a straight spore chain. Spores were visualized as elongated, rod-shaped, smooth surfaces with curved edges, as shown in Figure 1. When grown on different *Actinomyces*-specific media, *S. tauricus* displayed distinct phenotypic characteristics as shown in Table 1.

**Table 1:** Optimization of different growth media for *Streptomyces tauricus.*

Media	Growth	Front view	Rear view	Pigment	Spores
Sucrose peptone agar	Good	Creamish	Creamish	−	White
Glucose leucine agar	No	−	−	−	No
Nutrient agar	Good	Cream	Cream	−	−
Malt extract agar	Moderate	Cream	Cream	−	No
Yeast extract agar	Good	Cream	Cream	−	No
Starch casein nitrate agar	Excellent	White	Brown	+	Ash

Excellent growth was achieved on starch casein nitrate (SCN) agar, whereas moderate growth was observed on sucrose-peptone agar, yeast extract agar, and nutrient agar media. Moderate growth was seen on malt extract agar. No growth was seen on glucose-leucine agar. Yeast malt extract agar as the best growth medium for *S. tauricus* in another study (Rather et al., 2017).

In our previous report, when *S. tauricus* was subjected to the simple and rapid disruption method according to Bhaduri, it yielded significant intracellular extract (Bhaduri and Demchick, 1983).

**Figure 2:**
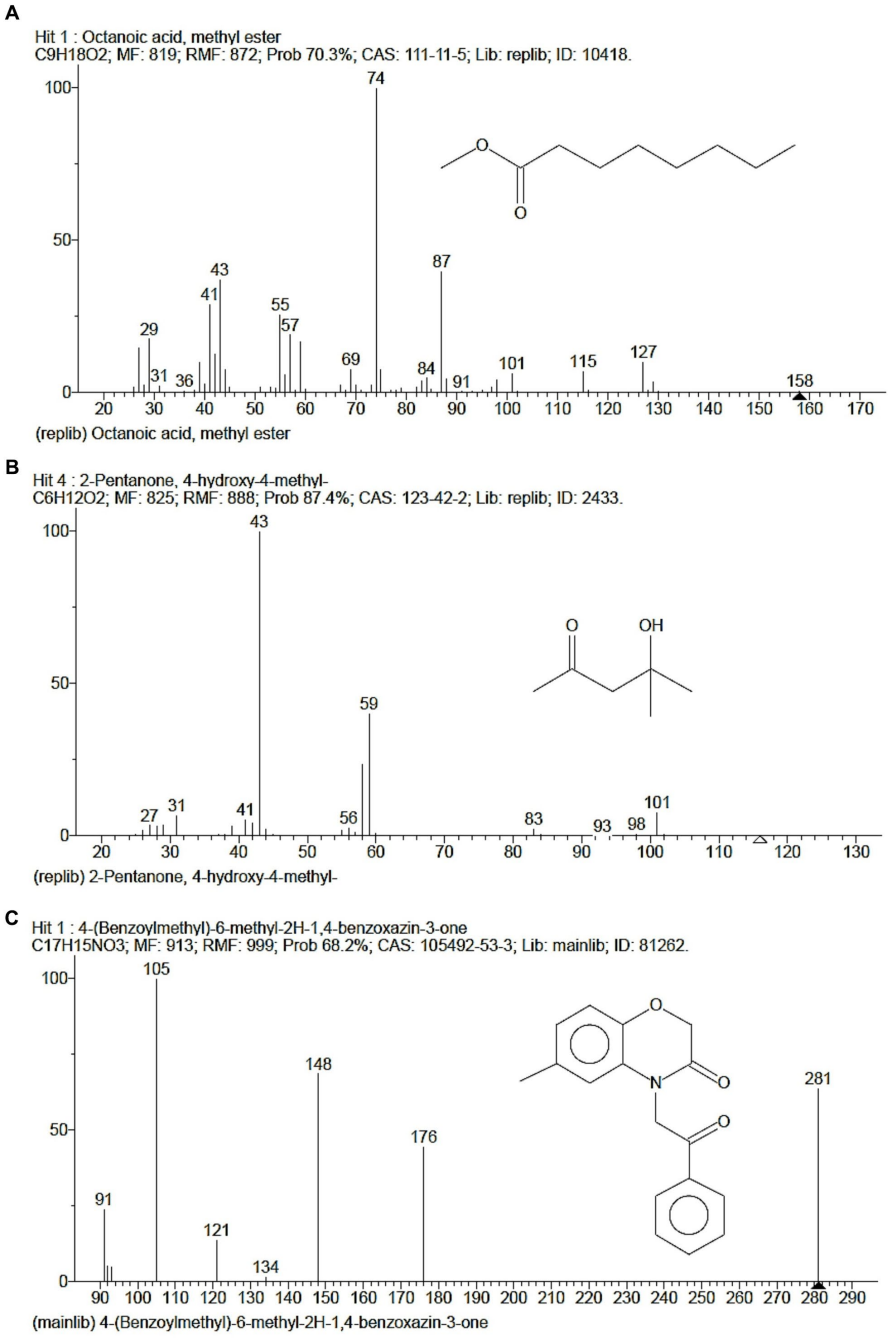
GC-MS depicted highest probable compounds. **(A)** Octanoic acid, methyl ester having 70% probability. **(B)** 2-Pentanone, 4-hydroxy-methyl having 87.4% probability. **(C)** 4-(Benzoylmethyl)-6-methyl-2H-1, 4-benzoxazin-3-one having 68.2% probability.

The GCMS studies depicted the presence of 98 bioactive compounds present in the intracellular extract of *S. tauricus* and the elution profile as shown in Supplementary Figure S1. GC–MS analysis revealed the most likely compounds, such as octanoic acid and methyl ester with 70% probability, 2-pentanone, 4-hydroxy-methyl with 87.4% probability, 4-(benzoylmethyl)-6-methyl-2H-1 with 68.2% probability, and 4-benzoxazin-3-one with 68.2% probability, as shown in Figure 2. All the compounds detected through GC-MS showed a low molecular weight below 1 kDa with various pharmacological applications. The majority of bioactive compounds have shown antimicrobial, enzyme inhibitors, activators, antioxidants, anti-inflammatory, anti-cancer, agrochemical, insecticide, anti-obese, and many other applications as listed in Supplementary Table S1.

**Figure 3:**
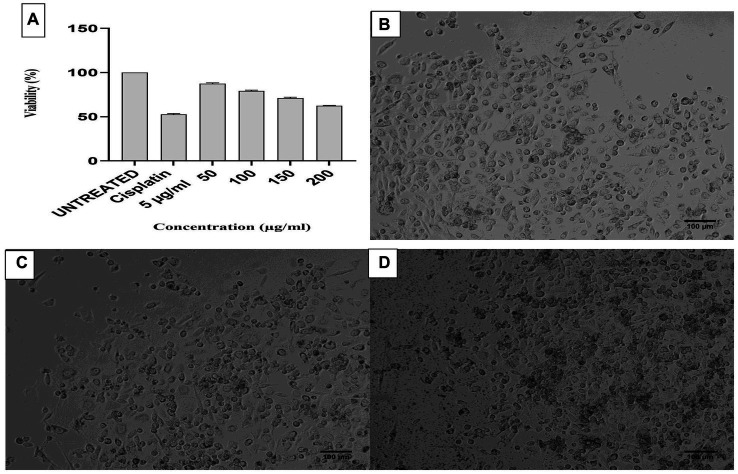
**(A)** Anticancer activity of *Streptomyces tauricus* strain protein. MTT assay performed by using prostate cancer PC3 cell line. **(B)** Untreated cells of PC3 cell line. **(C)** Standard cisplatin at 5 μg/mL. **(D)** 37.351% Anticancer activity of *S. tauricus* protein at 200 μg/mL.

The eluted peak fraction protein of *S. tauricus* showed significant activity for different protein concentrations, such as 87% cell viability for 50 μg of protein, 79% cell viability for 100 μg, 71% cell viability for 150 μg, and 62% cell viability for 200 μg against prostate cancer PC3 cell line comparison with standard drug cisplatin at 5 μg showed 53% cell viability, *p* > 0.05 statistically non-significant and error graph as shown in Figure 3.

A similar study reported that Actinomycin-D, a bioactive compound, was isolated from *S. tauricus,* characterized through LCMS, and showed effective antiproliferative activity against HeLa, PC3, THP-1, and Caco-2 cell lines (Rather et al., 2017). Another study reported that *S. variabilis is* able to produce bioactive compounds and showed increases in adipogenesis and glyceroneogenesis, exhibits anticancer and cytotoxic activity, and has purging properties in autologous bone marrow transplantation. It also alters the differentiation of human liposarcoma (Kesavan et al., 2014). Antimicrobial metabolites were also successfully isolated from Actinomyces grown on starch, casein, and nitrate agar in a marine salt pan (Ballav et al., 2015).

**Figure 4:**
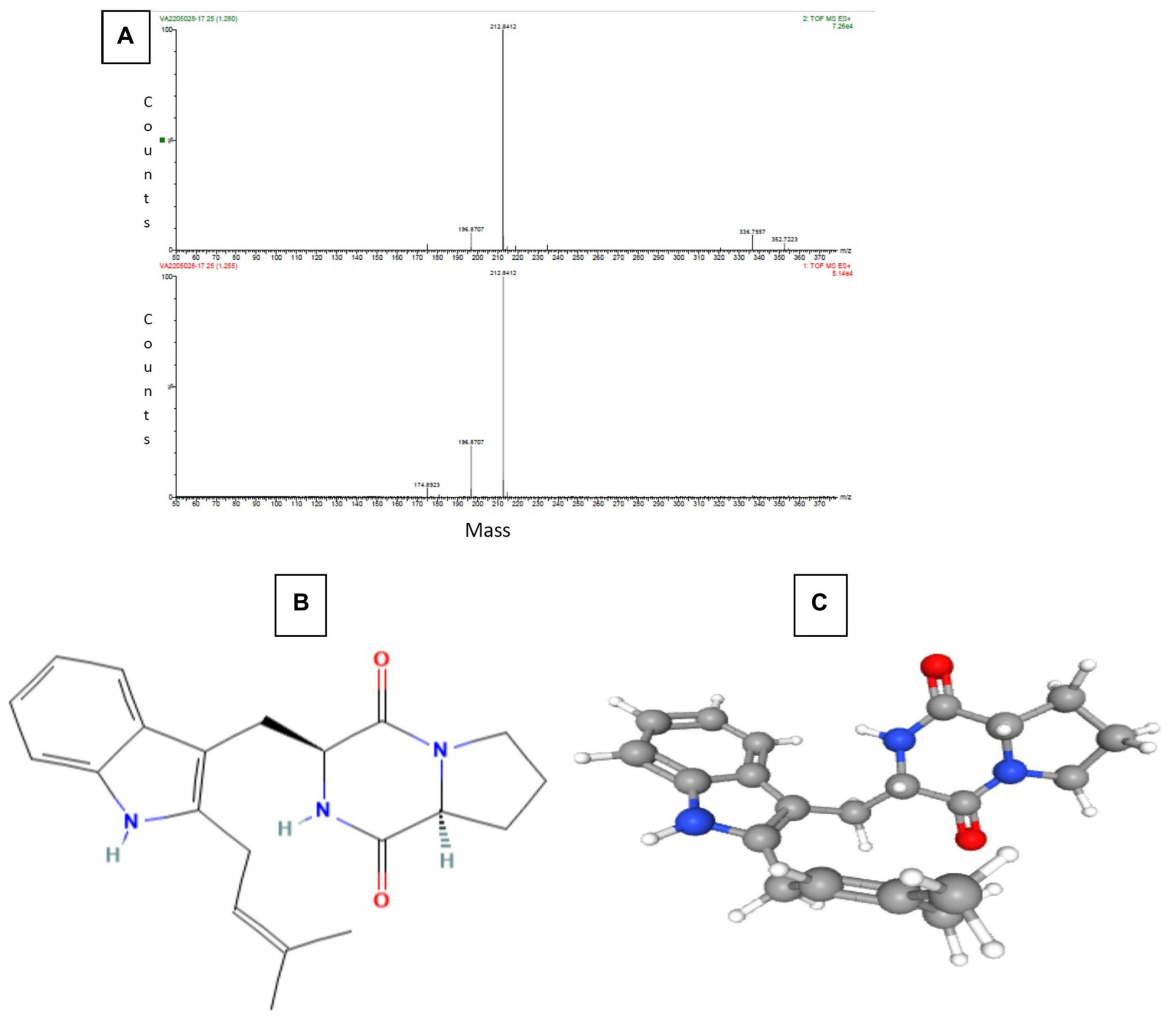
LC-MS analysis. **(A)** Mass confirmation of Tryprostatin B. **(B)** 2D structure of Tryptostatin B. **(C)** 3D structure of Tryptostatin B.

**Figure 5:**
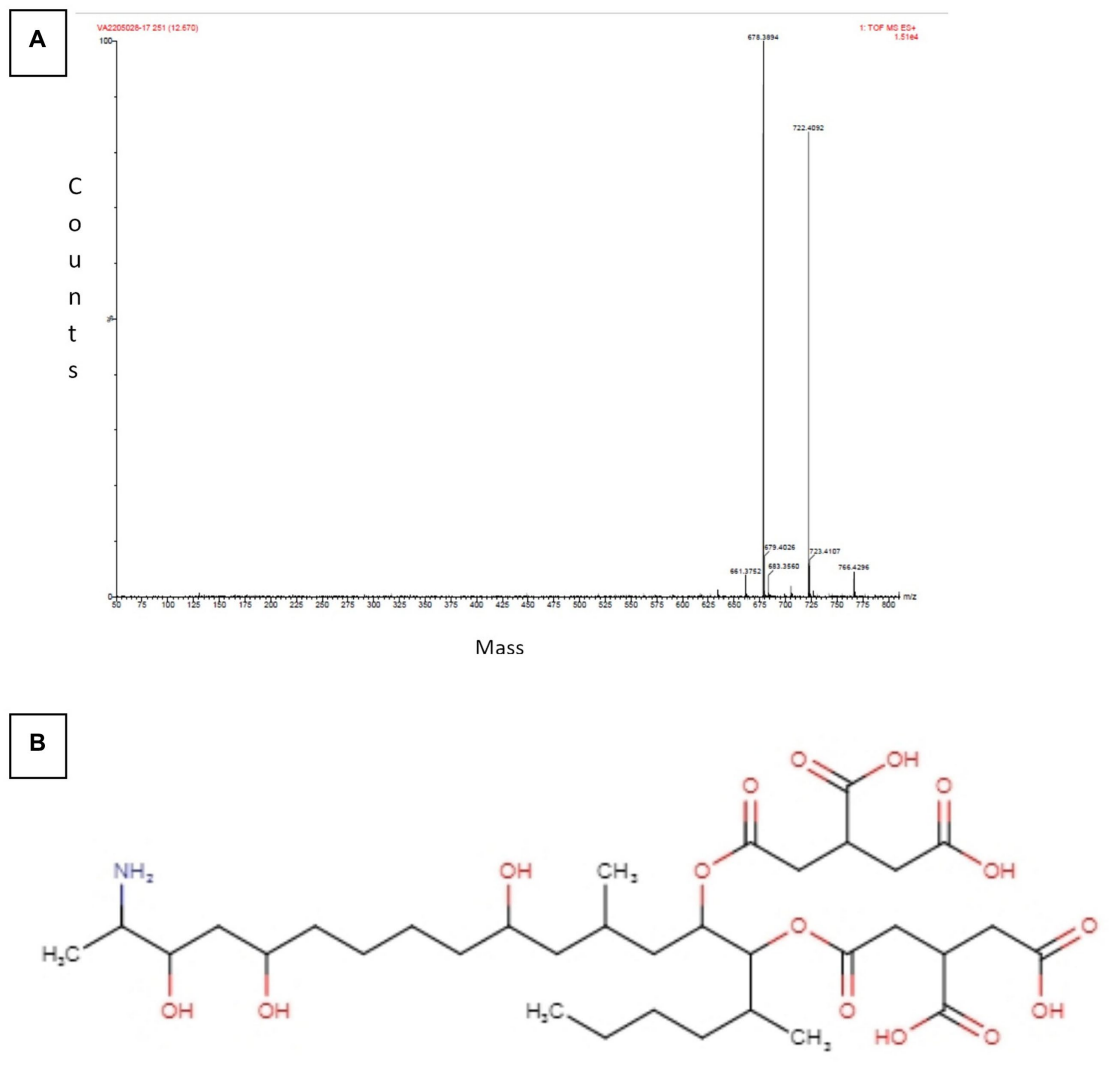
LC-MS analysis. **(A)** Mass confirmation of fumonisin B1. **(B)** 2D structure of fumonisin B1.

**Figure 6:**
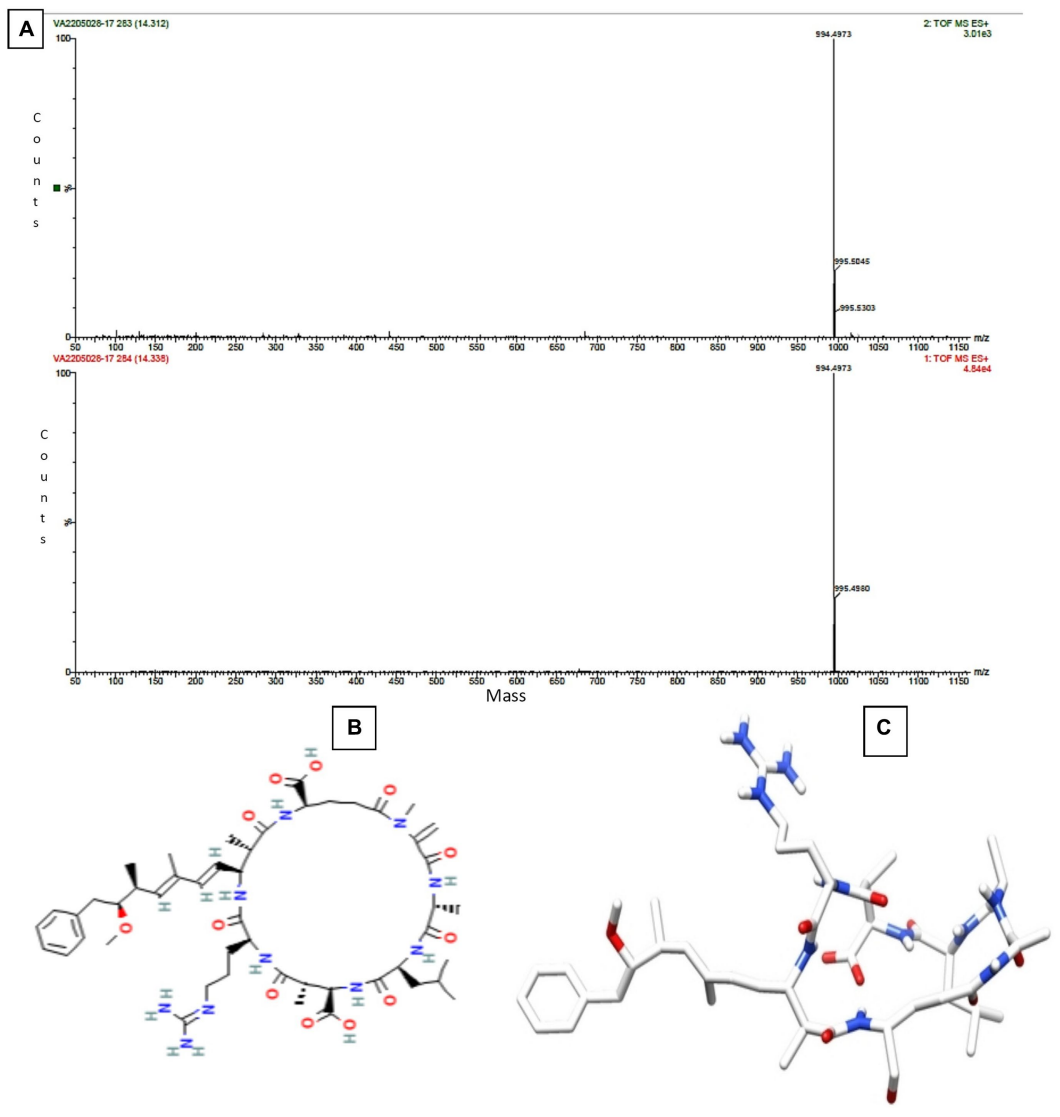
LC-MS analysis. **(A)** Mass confirmation of Microcystin LR. **(B)** 2D structure of Microcystin LR. **(C)** 3D structure of Microcystin LR.

**Figure 7:**
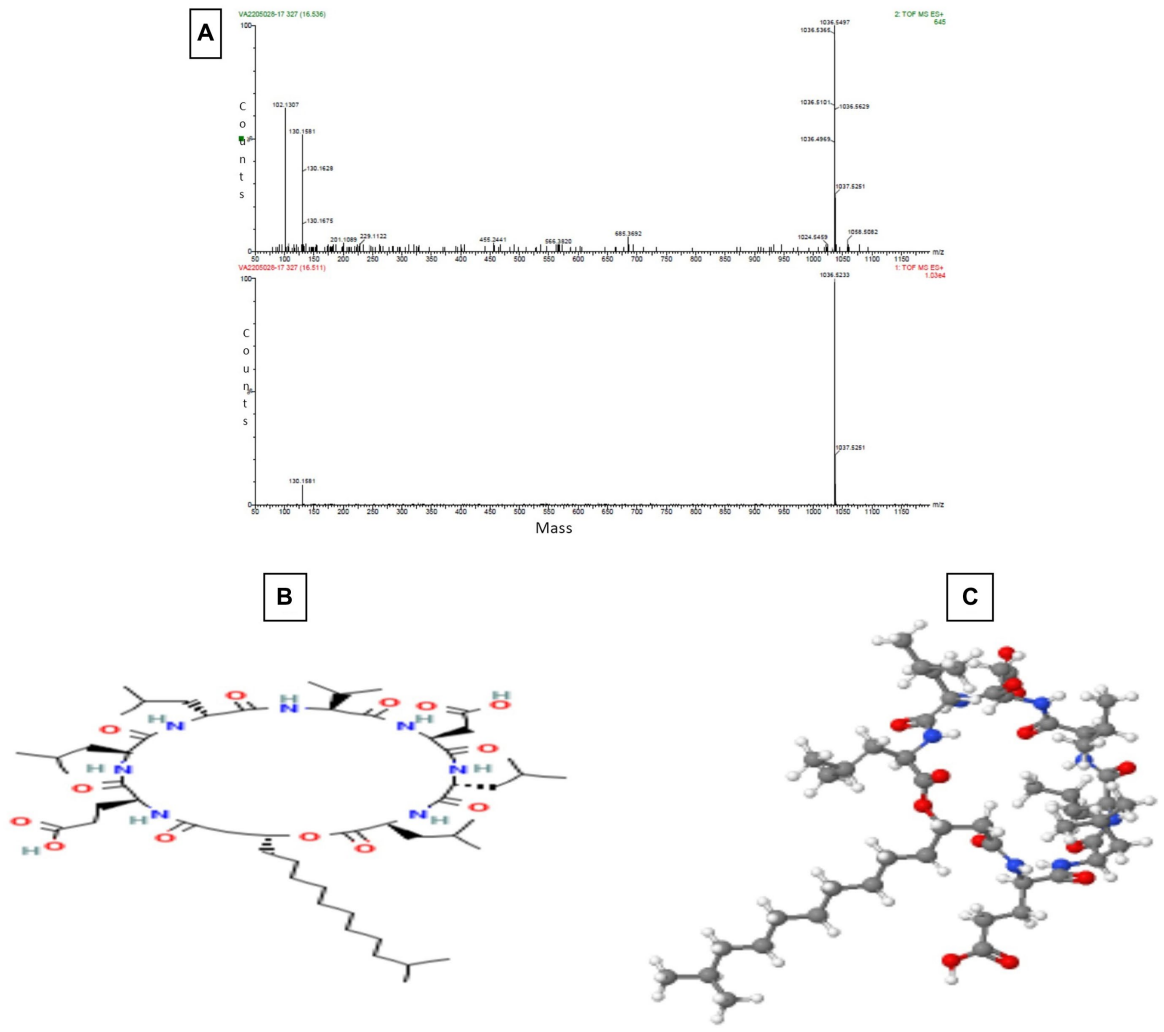
LC-MS Analysis **(A)** Mass confirmation of Surfactin C **(B)** 2D structure of Surfactin C **(C)** 3D structure of Surfactin C.

The Sephadex G-10 eluted peak protein fraction was further subjected to LCMS analysis. The LCMS analysis and elution profile, as shown in Supplementary Figure S2, revealed the detection of pharmacologically applicable bioactive peptide compounds. The elution peak from LCMS analysis and detection through the NIST computer library resulted in the identification of Tryprostatin B, fumonisin B1, Microcystin LR, and Surfactin C. Figure 4 depicts an LCMS analysis and comparison with the NIST library of Tryprostatin B with a 351 Da molecular weight structure and mass confirmation. The mass confirmation and structure of fumonisin B1 with a molecular weight of 721 Da are shown in Figure 5. Figure 6 depicts the structure and mass confirmation of Microcystin LR, which has a molecular weight of 994 Da. Figure 7 depicts Surfactin C with a molecular weight of 1,035 Da, structure, and mass confirmation. These bioactive molecules are well-known for their effective activity in various biological applications.

Tryprostatin B is an active peptide with varied biological properties, including anticancer, anti-mitotic, and potential *in vitro* and *in vivo* anticancer action. Brevianamide F and tryprostatin B are both cyclic dipeptides. Tryprostatin B differs from Brevianamide F by having a prenyl group added to the second position of the indole ring (Yamakawa et al., 2010; Huisman et al., 2019). Deoxy brevianamide E, also known as tryprostatin B, is a crucial step in the biosynthesis of the anti-mitotic pathway (Cytotoxic activity). Studies in biochemistry and genetics have revealed the roles of biosynthetic genes and isolated significant intermediates and shunt products from these processes (Pan et al., 2014).

The enzyme ceramide synthase, also known as sphingosine N-acyltransferase, which acylates sphingoid bases, is inhibited by fumonisin B1. This prevents the production of ceramide in two different ways. It prevents the production of new sphinganine, fatty acyl-CoA, and sphingosine, which are formed when ceramidase breaks down ceramide (Merrill et al., 2001). The cytotoxic and growth-inhibitory properties of sphinganine and sphingosine are present. These sphingoid bases also trigger apoptosis. Increased apoptosis appears to be crucial for the harmful effects, including the formation of tumors (Zentai et al., 2019).

In freshwater lakes and rivers throughout the world, *Microcystis aeruginosa* is the primary producer of microcystins (MCs), the most pervasive cyanobacterial toxins. The seven-membered peptide ring of MCs, a complicated class of monocyclic heptapeptides with considerable hepatotoxicity, is made up of two protein amino acids and five non protein amino acids. Due to the different proportions of the two variable protein amino acids, over 100 MC variants have been discovered thus far and named according to the variable amino acids that complete their structures. MC-LR, MC-RR, MC-YR, and MC-LA (L, leucine; R, arginine; Y, tyrosine; and A, alanine) are the most prevalent and abundant MCs, with MC-LR being the most researched and hazardous of the four. Hepatotoxins called microcystins strongly bind to serine/threonine protein phosphatases (PPs), enzymes that can remove phosphate from proteins in a variety of metabolic pathways (Li et al., 2017; Wu et al., 2019).

Numerous Bacillus strains produce the highly effective bio-surfactant lipo-peptide Surfactin C, which has hemolytic, antiviral, anticancer, and antibacterial properties. This anionic cyclic lipopeptide is composed of a heptapeptide linked to a-hydroxy fatty acid. Surfactin permeabilizes and perturbs target cells as a result of integrating into the phospholipid bilayer due to its amphipathic nature. As a result of the rise in antibiotic resistance as well as several noteworthy Surfactin activities, it is thought to be a potential chemical for treating a variety of health-related problems (Seydlová and Svobodová, 2008).

## Conclusion

The present study is illustrative for exploring untapped mangrove habitats in the Mangalore region of Karnataka. The *S. tauricus* spores were visualized as elongated, rod-shaped, smooth surfaces with curved edges. The intracellular extract was purified and characterized using GCMS and LCMS analysis reveals the peptides having molecular weight of <1 kDa (Tryprostatin B, Fumonisin B1, Microcystin LR, and Surfactin C) is an efficient microbe to produce bioactive peptides for both antimicrobial and anticancer activity. Hence, this study supports and proves that the genus *Streptomyces* is an effective microbial group for the isolation source of bioactive peptides to treat multidrug-resistant pathogens, anticancer activity on prostate cancer cell lines, and various other related ailments.

## Data availability statement

The original contributions presented in the study are publicly available. This data can be found here: GenBank repository, accession number MW785875.

## Author contributions

YK and MK contributed to the design of the experiments. YK performed the experiments. MK, SK, and KR guided in performing the experiments. YK and MM analyzed the results and wrote the manuscript. YK, MK, SK, KR, SS, SA, OA, and MM wrote and reviewed the manuscript. All authors contributed to the article and approved the submitted version.

## Funding

This work was supported by the Science and Engineering Research Board, DST, Govt. of India, and Vision Group of Science and Technology Govt. of Karnataka by providing financial and equipment grants.

## Conflict of interest

The authors declare that the research was conducted in the absence of any commercial or financial relationships that could be construed as a potential conflict of interest.

## Publisher’s note

All claims expressed in this article are solely those of the authors and do not necessarily represent those of their affiliated organizations, or those of the publisher, the editors and the reviewers. Any product that may be evaluated in this article, or claim that may be made by its manufacturer, is not guaranteed or endorsed by the publisher.

## References

[ref1] Abd-Elnaby H. Abo-Elala G. Abdel-Raouf U. Abd-elwahab A. Hamed M. (2016). Antibacterial and anticancer activity of marine *Streptomyces parvus*: optimization and application. Biotechnol Biotechnol Equipment 30, 180–191. doi: 10.1080/13102818.2015.1086280

[ref2] Alongi D. M. (2015). The impact of climate change on mangrove forests. Curr Clim Change Rep 1, 30–39. doi: 10.1007/s40641-015-0002-x

[ref3] Ballav S. Kerkar S. Thomas S. Augustine N. (2015). Halophilic and halotolerant actinomycetes from a marine saltern of Goa, India producing anti-bacterial metabolites. J. Biosci. Bioeng. 119, 323–330. doi: 10.1016/j.jbiosc.2014.08.017, PMID: 25449757

[ref4] Bhaduri S. Demchick P. H. (1983). Simple and rapid method for disruption of bacteria for protein studies. Appl. Environ. Microbiol. 46, 941–943. doi: 10.1128/aem.46.4.941-943.1983, PMID: 6639038 PMC239492

[ref5] Burckhardt R. M. Van Drisse C. M. Tucker A. C. Escalante-Semerena J. C. (2020). New AMP-forming acid:CoA ligases from Streptomyces lividans, some of which are posttranslationally regulated by reversible lysine acetylation. Mol. Microbiol. 113, 253–269. doi: 10.1111/mmi.14414, PMID: 31677300 PMC7007339

[ref6] Chater K. F. (2013). “Streptomyces” in Brenner’s encyclopedia of genetics. 2nd Edn. ed. J. Parker (Cambridge, Massachusetts, United States: Elsevier), 565–567.

[ref7] Desouky S. E. Shojima A. Singh R. P. Matsufuji T. Igarashi Y. Suzuki T.. (2015). Cyclodepsipeptides produced by actinomycetes inhibit cyclic-peptide-mediated quorum sensing in gram-positive bacteria. FEMS Microbiol. Lett. 362:fnv109. doi: 10.1093/femsle/fnv109, PMID: 26149266

[ref8] Fernebro J. (2011). Fighting bacterial infections-future treatment options. Drug Resist. Updat. 14, 125–139. doi: 10.1016/j.drup.2011.02.001, PMID: 21367651

[ref9] Huisman M. Rahaman M. Asad S. Oehm S. Novin S. Rheingold A. L.. (2019). Total synthesis of Tryprostatin B: synthesis and asymmetric phase-transfer-catalyzed reaction of Prenylated Gramine salt. Org. Lett. 21, 134–137. doi: 10.1021/acs.orglett.8b03593, PMID: 30561217

[ref10] Janardhan A. Kumar A. P. Viswanath B. Saigopal D. V. Narasimha G. (2014). Production of bioactive compounds by actinomycetes and their antioxidant properties. Biotechnol. Res. Int. 2014:217030. doi: 10.1155/2014/217030 24790761 PMC3984769

[ref11] Karthik Y. Ishwara Kalyani M. Krishnappa S. Devappa R. Anjali Goud C. Ramakrishna K.. (2023). Antiproliferative activity of antimicrobial peptides and bioactive compounds from the mangrove Glutamicibacter mysorens. Front. Microbiol. 14:826. doi: 10.3389/fmicb.2023.1096826 PMC9982118 36876075

[ref12] Karthik Y. Kalyani M. I. (2022). Occurrence of *Streptomyces tauricus* in mangrove soil of Mangalore region in Dakshina Kannada as a source for antimicrobial peptide. J. Basic Microbiol. 63, 389–403. doi: 10.1002/jobm.202200108, PMID: 35876342

[ref13] Karthik Y. Kalyani M. I. Ishwar M.. (2020). Cytotoxic and antimicrobial activities of microbial proteins from mangrove soil actinomycetes of Mangalore, Dakshina Kannada. Biomedicine 40, 59–67. doi: 10.51248/.v40i1.104

[ref14] Kesavan S. S. S. Bavanilatha M. Vijyalakshmi R. Hemalatha S. (2014). Analysis of bioactive constituents from a new *Streptomyces Variabilis* strain SU5 by gas chromatography-mass spectrometry. International. J. Pharm. Pharm. Sci. 6, 224–226.

[ref15] Li J. Li R. Li J. (2017). Current research scenario for microcystins biodegradation – a review on fundamental knowledge, application prospects and challenges. Sci. Total Environ. 595, 615–632. doi: 10.1016/j.scitotenv.2017.03.285, PMID: 28407581

[ref16] Merrill A. H. Sullards M. C. Wang E. Voss K. A. Riley R. T. (2001). Sphingolipid metabolism: roles in signal transduction and disruption by Fumonisins. Environ. Health Perspect. 109, 283–289. doi: 10.2307/3435020 PMC1240677 11359697

[ref17] Mohan Y. Sirisha B. Haritha R. Ramana T. (2013). Selective screening, isolation and characterization of antimicrobial agents from marine actinomycetes. Int J Pharm Pharm Sci 5, 443–449.

[ref18] Nishioka K. Katayama I. (1978). Angiogenic activity in culture supernatant of antigen-stimulated lymph node cells. J. Pathol. 126, 63–69. doi: 10.1002/path.1711260202, PMID: 739288

[ref19] Pan L.-L. Yang Y. Merz K. M. (2014). Origin of product selectivity in a Prenyl transfer reaction from the same intermediate: exploration of multiple Ftm PT1-catalyzed Prenyl transfer pathways. Biochemistry 53, 6126–6138. doi: 10.1021/bi500747z, PMID: 25188320 PMC4179596

[ref20] Rather S. A. Shah A. M. Ali S. A. Dar R. A. Rah B. Ali A.. (2017). Isolation and characterization of *Streptomyces tauricus* from Thajiwas glacier—a new source of actinomycin-D. Med. Chem. Res. 26, 1897–1902. doi: 10.1007/s00044-017-1842-9

[ref21] Renner M. K. Shen Y.-C. Cheng X.-C. Jensen P. R. Frankmoelle W. Kauffman C. A.. (1999). Cyclomarins a−C, new Antiinflammatory cyclic peptides produced by a marine bacterium (Streptomyces sp.). J. Am. Chem. Soc. 121, 11273–11276. doi: 10.1021/ja992482o

[ref22] Sapkota A. Thapa A. Budhathoki A. Sainju M. Shrestha P. Aryal S. (2020). Isolation, characterization, and screening of antimicrobial-producing Actinomycetes from soil samples. Int J Microbiol 2020, 1–7. doi: 10.1155/2020/2716584, PMID: 32300363 PMC7139855

[ref23] Seydlová G. Svobodová J. (2008). Review of surfactin chemical properties and the potential biomedical applications. Cent. Eur. J. Med 3, 123–133. doi: 10.2478/s11536-008-0002-5

[ref24] Sridevi V. Priyadarshini P. S. Gautam Y. A. (2015). Selection of marine actinomycetes with bioactive potential isolated from sediments of bay of Bengal and characterization of promising isolate, ABT-103. Microbiol Res J Int, 10, 1–9. doi: 10.9734/BMRJ/2015/20132

[ref25] Wang C. Lu Y. Cao S. (2020). Antimicrobial compounds from marine actinomycetes. Arch. Pharm. Res. 43, 677–704. doi: 10.1007/s12272-020-01251-0, PMID: 32691395 PMC7703873

[ref26] Wu J. Chen D. Wu J. Chu X. Yang Y. Fang L.. (2021). Comparative transcriptome analysis demonstrates the positive effect of the cyclic AMP receptor protein Crp on Daptomycin biosynthesis in *Streptomyces roseosporus*. Front. Bioeng. Biotechnol. 9:e618029. doi: 10.3389/fbioe.2021.618029, PMID: 34150723 PMC8212052

[ref27] Wu J. Wang X. Wang Q. Lou Z. Li S. Zhu Y.. (2019). Nanomaterials with enzyme-like characteristics (nanozymes): next-generation artificial enzymes (II). Chem. Soc. Rev. 48, 1004–1076. doi: 10.1039/C8CS00457A, PMID: 30534770

[ref28] Yamakawa T. Ideue E. Shimokawa J. Fukuyama T. (2010). Total synthesis of Tryprostatins A and B. Angew. Chem. Int. Ed. Engl. 49, 9262–9265. doi: 10.1002/anie.201004963 21031390

[ref29] Zentai A. Szeitzné-Szabó M. Mihucz G. Szeli N. Szabó A. Kovács M. (2019). Occurrence and risk assessment of Fumonisin B1 and B2 mycotoxins in maize-based food products in Hungary. Toxins 11:709. doi: 10.3390/toxins11120709, PMID: 31817520 PMC6950155

