# Supplementary figures and images for: Promising bioactive metabolites of mangrove inhabitant Streptomyces tauricus and prostate cancer PC3 cell inhibition by antimicrobial peptides
Source: Front Microbiol. 2023 Jun 16;14:1152985. doi: 10.3389/fmicb.2023.1152985 (PMC10312093; doi:10.3389/fmicb.2023.1152985)

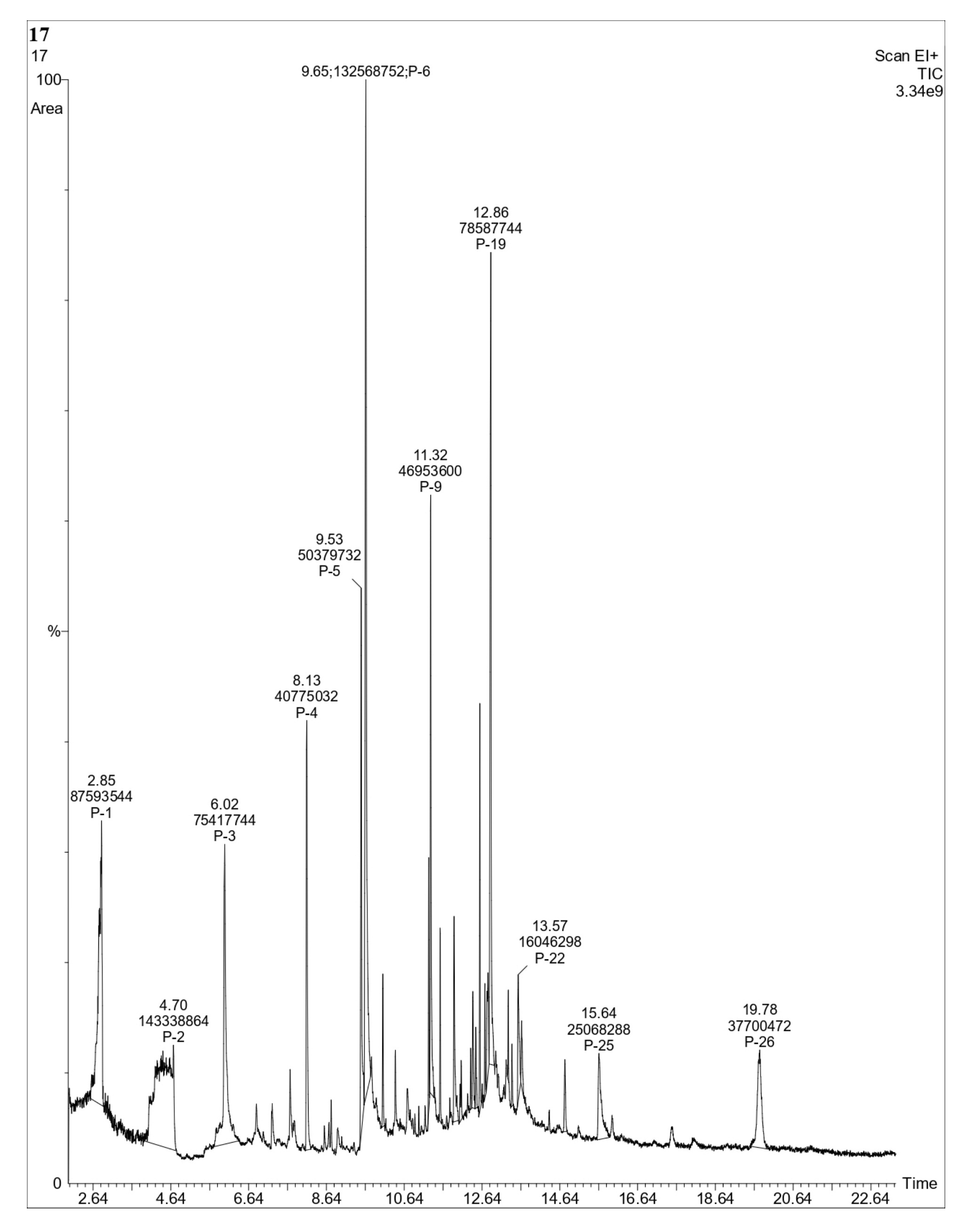

Supplement: Supplementary file 1 [file Image_1.JPEG]

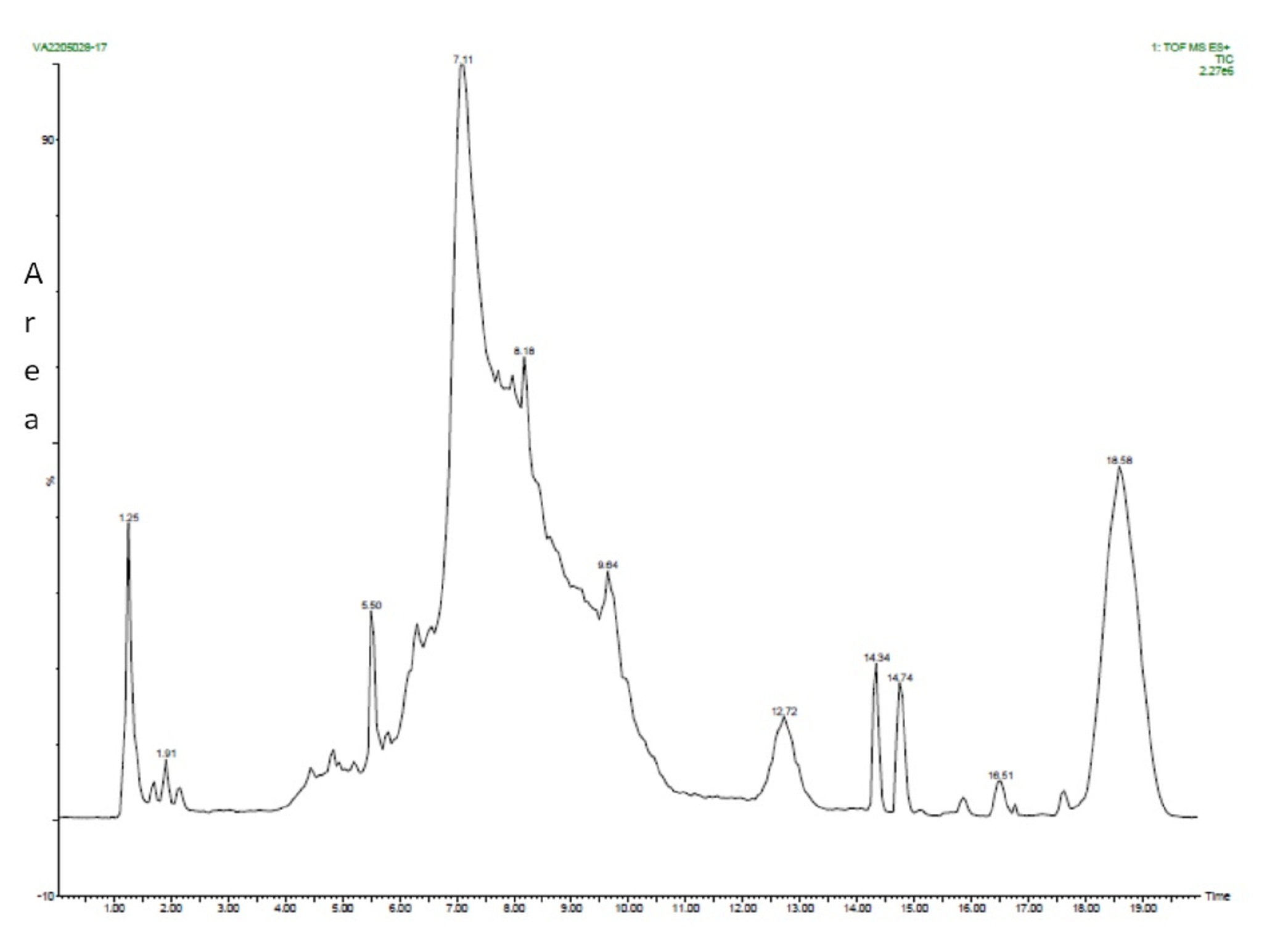

Supplement: Supplementary file 2 [file Image_2.JPEG]
