# Supplementary material for: Promising bioactive metabolites of mangrove inhabitant Streptomyces tauricus and prostate cancer PC3 cell inhibition by antimicrobial peptides
Source: Front Microbiol. 2023 Jun 16;14:1152985. doi: 10.3389/fmicb.2023.1152985 (PMC10312093; doi:10.3389/fmicb.2023.1152985)
Supplement: Supplementary file 3 [file Table_1.docx]

**Table S1.** List of GC-MS analysis of bioactive compounds from *S. tauricus* intracellular extract

| S. No. | R.T (min) | Compound name | Activity/Application | Molecular  Formula | Molecular  weight (g/mol) | Area % | References |
| --- | --- | --- | --- | --- | --- | --- | --- |
| 1 | 4.5 | 2-Pentanone, 4-hydroxy-4-methyl- | paint thinner, wood colorant, rust remover | C_6_H_12_O_2_ | 116 | 0.9 | Qiu et al. (2019) |
| 2 |  | tert-Butyl Hydroperoxide | Oxidant | C_4_H_10_O_2_ | 90 |  | Gad (2014) |
| 3 |  | 1,3-Dioxolane-2-methanol, 2,4-dimethyl- | Chlorinating agent | C_6_H_12_O_3_ | 132 |  | Simon and Losada (2008); Fuentes et al. (2016) |
| 4 |  | 2-Propanol, 2-nitroso-, acetate | Cosmetics | C_5_H_9_NO_3_ | 131 |  | Lemieux and Nagabhushan (1968) |
| 5 |  | 2-Hexanone, 4-methyl- | Paints | C_7_H_14_O | 114 |  | Rebbert and Ausloos (1962) |
| 6 |  | 2-Acetoxyisobutyryl chloride | Epoxides synthesis | C_6_H_9_ClO_3_ | 164 |  | Zibuck (2001) |
| 7 | 6.0 | Octanoic acid, methyl ester | Oxidation | C_9_H_18_O_2_ | 158 | 8.6 | Schwabe et al. (1964) |
| 8 |  | Undecanoic acid, 2-methyl- | Antifungal | C_12_H_24_O_2_ | 200 |  | Rossi et al. (2021) |
| 9 |  | Methyl 6-methyl heptanoate | Biomolecule synthesis | C_9_H_18_O_2_ | 158 |  | Kroumova and Wagner (2003) |
| 10 |  | Decanoic acid, methyl ester | Antibacterial | C_11_H_22_O_2_ | 186 |  | Damiano et al. (2020) |
| 11 |  | Cyclopentaneundecanoic acid, methyl ester | Antioxidant and Antibacterial | C_17_H_32_O_2_ | 268 |  | Daniels and Temikotan (2021) |
| 12 | 6.8 | 1-Octanol, 2,7-dimethyl- | Antioxidant, hepatoprotective and anti-inflammatory | C_10_H_22_O | 158 |  | Abdillah et al. (2015) |
| 13 |  | Carbonic acid, prop-1-en-2-yl undecyl ester | Beverages production | C_15_H_28_O_3_ | 256 |  | Millero et al. (2006) |
| 14 |  | Hydroxylamine, O-decyl- | Reducing agent | C_10_H_23_NO | 173 |  | Gad (2005) |
| 15 |  | 1-Decanol, 2-ethyl- | Surfactant | C_12_H_26_O | 186 |  | Achimón et al. (2022) |
| 16 |  | 1-Decanol, 2-methyl- | Lubricants, Plasticizers | C_11_H_24_O | 172 |  | Halling et al. (1998) |
| 17 |  | Trichloroaceticacid, decyl ester | Disinfectant | C_12_H_21_Cl_3_O_2_ | 302 |  | Anand et al. (2014) |
| 18 |  | 1-Heptanol, 2-propyl- | Pheromone | C_10_H_22_O | 158 |  | Francke and Schulz (1999) |
| 19 |  | Octadecane, 1-(ethenyloxy)- | Anti-corrosion | C_20_H_40_O | 296 |  | Zeitoun et al. (2021) |
| 20 |  | Carbonic acid, decyl prop-1-en-2-yl ester | Beverages production | C_14_H_26_O_3_ | 242 |  | Millero et al. (2006) |
| 21 | 7.2 | 1,7-Octanediol, 3,7-dimethyl- | Polymer | C_10_H_22_O_2_ | 174 | 8.6 | Reddy and Ananthaprasad (2021); |
| 22 |  | Octanoic acid, 7-oxo- | Antibacterial | C_8_H_14_O_3_ | 158 |  | Schwabe et al. (1964) |
| 23 |  | 1,8-Nonanediol, 8-methyl- | Agrochemicals | C_10_H_22_O2 | 174 |  | Kula et al. (2001) |
| 24 |  | 7-Octen-2-ol, 2,6-dimethyl- | Cosmetics | C_10_H_20_O | 156 |  | Ham and Raymond Wells (2009) |
| 25 |  | Methyl 6-oxoheptanoate | Antibacterial | C_8_H_14_O_3_ | 158 |  | Idan et al. (2015) |
| 26 |  | 3-Heptanol, 4-methyl- | Therapeutics | C_8_H_18_O | 130 |  | Ley and Madin (1991) |
| 27 |  | 4-Heptanone, 2,3:5,6-diepoxy-2,6-dimethyl- | Oxidant | C_9_H_14_O_3_ | 170 |  | Ley and Madin (1991) |
| 28 |  | 3-Tridecanol | Lubricant | C_13_H_28_O | 200 |  | Chagnes et al. (2010) |
| 29 |  | 2-Dodecanone | Insecticide | C_12_H_24_O | 184 |  | Wang et al. (2019) |
| 30 | 7.7 | Succinic acid, 2-methylpent-3-yl pentafluorobenzyl ester | Antioxidant | C_17_H_19_F_5_O_4_ | 382 | 8.6 | Cullere et al. (2004) |
| 31 |  | 1,1'-Biphenyl, 2-iodo- | Substrate | C_12_H_9_I | 280 |  | Fang et al. (2021) |
| 32 |  | Benzamide, N-(1,4,6-trimethyl-1H-pyrazolo[3,4-b]pyridin-3-yl)- | Substrate | C_16_H_16_N_4_O | 280 |  | Jachak et al. (2006) |
| 33 |  | 4-[N'-(4-Methoxy-benzoyl)-hydrazino]-4-oxo-butyric acid methyl ester | Antibacterial | C_13_H_16_N_2_O_5_ | 280 |  | EL-Hashash et al. (2014) |
| 34 |  | Dibenzo[a,c]phenazine | Fluorochrome | C_20_H_12_N_2_ | 280 |  | Xie et al. (2019) |
| 35 |  | Benzofuro[3,2-d]pyrimidine, 4-(2-pyridylthio)- | Therapeutic | C_15_H_9_N_3_OS | 279 |  | Campos et al. (2022) |
| 36 |  | (9E)-Styrylanthracene | Luminophore | C_22_H_16_ | 280 |  | Zhang et al. (2017) |
| 37 |  | 1H-Purine-2,6-dione,3,7-dihydro-3-methyl-7-carboxymethyl-8-n-butyl | Anti-inflammatory | C_12_H_16_N_4_O_4_ | 280 |  | Abou-Ghadir et al. (2014) |
| 38 |  | Methyl 2-phenyl-2,3-epoxyindan-1-one-3-carboxylate | Catalyst | C_17_H_12_O_4_ | 280 |  | Godwin et al. (2012) |
| 39 |  | Propyl N-(heptafluorobutyryl)pyroglutamate | Metabolite | C_12_H_12_F_7_NO_4_ | 367 |  | (Hušek et al. (2016) |
| 40 | 8.7 | Z,Z-2,5-Pentadecadien-1-ol | Pharmacological | C_15_H_28_O | 224 | 6.8 | Ganesh and Mohankumar (2017) |
| 41 |  | 10-Octadecenal | Adjuvant/ pheromones | C_18_H_34_O | 266 |  | Gil et al. (1995) |
| 42 |  | 3-(Prop-2-enoyloxy)tetradecane | Phytoconstituent | C_17_H_32_O_2_ | 268 |  | Ezekwe et al. (2020) |
| 43 |  | l-Gala-l-ido-octose | Neuritogenic | C_8_H_16_O_8_ | 240 |  | Jahan et al. (2020) |
| 44 |  | 5-(Prop-2-enoyloxy)pentadecane | Antimicrobial | C_18_H_34_O_2_ | 282 |  | Xue et al. (2017); Gadhi et al. (2019) |
| 45 |  | 2-Cyclopropylcarbonyloxytridecane | aphrodisiac, anti-inflammatory, antihypertensive | C_17_H_32_O_2_ | 268 |  | Bhargawanet al. (2016) |
| 46 |  | Imidazole, 2-amino-5-[(2-carboxy)vinyl]- | Therapeutic | C_6_H_7_N_3_O_2_ | 153 |  | Shalini et al. (2010) |
| 47 |  | 4-Cyclopropylcarbonyloxytetradecane | Cytotoxic and Antibacterial | C_18_H_34_O_2_ | 282 |  | Talbaoui et al. (2020) |
| 48 | 9.5 | 1-Chloroundecane | Precursor for fatty acid synthesis | C_11_H_23_Cl | 190 | 14.7 | Gensler and Thomas (1952) |
| 49 |  | Dodecane, 1-chloro- | Hydrocarbon | C_12_H_25_Cl | 204 |  | Moldoveanu (2019) |
| 50 |  | Tetradecane, 1-chloro- | Chlorination | C_14_H_29_Cl | 232 |  | Assassi et al. (2005) |
| 51 |  | Nonane, 1-chloro- | Hydrocarbon | C_9_H_19_Cl | 162 |  | Moldoveanu (2019) |
| 52 | 10.0 | Benzene, 1,4-bis(trifluoromethyl)- | Fluorochrome | C_8_H_4_F_6_ | 214 | 1.7 | Skhirtladze et al. (2022) |
| 53 |  | Pyrimidine, 4,5-diamino-6-chloro-2-(trifluoromethyl)- | Transcriptional activator | C_5_H_4_ClF_3_N_4_ | 212 |  | Palanki et al. (2000) |
| 54 |  | 1H-Imidazole, 1-(2,2,3,3,3-pentafluoro-1-oxopropyl)- | Anticancer | C_6_H_3_F_5_N_2_O | 214 |  | Zhang et al. (2014) |
| 55 |  | Sulfaguanidine | Enzyme inhibitor | C_7_H_10_N_4_O_2_S | 214 |  | Akocak et al. (2021) |
| 56 |  | Anthracene, 2-chloro- | Antibacterial | C_14_H_9_Cl | 212 |  | de Bony et al. (1984) |
| 57 |  | Ethyl iodoacetate | Enzyme activator | C_4_H_7_IO_2_ | 214 |  | Tanaka and Hayashi (2008) |
| 58 |  | 8-Methyl-4-(1-pyrrolidinyl)pyrido[3,2-c]pyridazine | Cancer therapies | C_12_H_14_N_4_ | 214 |  | Jubete et al. (2019) |
| 59 |  | [1,1'-Biphenyl]-4-carboxylic acid, 4'-hydroxy- | Precursor for synthesis of bioactive molecules | C_13_H_10_O_3_ | 214 |  | Patel et al. (2009) |
| 60 |  | Benzoic acid, 2-(1,2,4-triazol-3-yl-aminocarbonyl)- | Breast and prostate cancer therapy | C_10_H_8_N_4_O_3_ | 232 |  | Jamieson et al. (2012) |
| 61 | 10.4 | 5-Amino-2-methoxy-4-(1H-1,2,3,4-tetrazol-5-yl)phenol | Antimicrobial | C_8_H_9_N_5_O_2_ | 207 | 1.7 | Arulmurugan and Kavitha (2010) |
| 62 |  | 4H-Pyrido[1,2-a]pyrimidine-3-carboxamide, 6,7,8,9-tetrahydro-6-methyl-4-oxo- | Antimicrobial and antitumor | C_10_H_13_N_3_O_2_ | 207 |  | Al-Taisan et al. (2010) |
| 63 |  | 1-Adamantanecarboxamide, N,N-dimethyl- | Anticancer | C_13_H_21_NO | 207 |  | Su et al. (2012) |
| 64 |  | trans-4-Ethoxy-β-methyl-β-nitrostyrene | Cardiovascular therapy | C_11_H_13_NO_3_ | 207 |  | Alves-Santos et al. (2019) |
| 65 |  | Pent-3-yn-2-ol, 2-cyclopropyl-5-(1-piperidyl)- | Anti-inflammatory | C_13_H_21_NO | 207 |  | Alam et al. (2020) |
| 66 |  | Carbamic acid, 4-methoxyphenyl-, allylester | Catalytic activity | C_11_H_13_NO_3_ | 207 |  | Anderson et al. (2005) |
| 67 |  | Thiophen-2-methylamine, N-(2-fluorophenyl)- | Catalytic activity | C_11_H_10_FNS | 207 |  | HasanTanak et al. (2020) |
| 68 |  | 2-(1-Piperidino)-3-nitropyridine | Antimicrobial | C_10_H_13_N_3_O_2_ | 207 |  | Sivaprakash et al. (2019) |
| 69 |  | Benzoic acid, 4-amino-, pentyl ester | Cytotoxicity | C_12_H_17_NO_2_ | 207 |  | Krátký et al. (2019) |
| 70 | 11.5 | 4-(Benzoylmethyl)-6-methyl-2H-1,4-benzoxazin-3-one | Antimicrobial | C_17_H_15_NO_3_ | 281 |  | Ozden et al. (2000) |
| 71 |  | Adenine, N4-pentafluoropropionyl- | Oxidization | C_8_H_4_F_5_N_5_O | 281 | 1.5 | Tsunoda et al. (2011) |
| 72 |  | 2-Furancarboxylic acid, N'-[(8-hydroxy-5-quinolinyl)methylidene]hydrazide | Antioxidant | C_15_H_11_N_3_O_3_ | 281 |  | Gülerman et al. (2000) |
| 73 |  | 1-Phenyl-4-(trifluoromethyl)-1H,4H,5H,6H,7H-pyrazolo[3,4-b]pyridin-6-one | Antiproliferative | C_13_H_10_F_3_N_3_O | 281 |  | Martín-Acosta et al. (2021) |
| 74 |  | Acetamide, 2-(2,4-difluorophenoxy)-N-(4-fluorophenyl)- | Inhibitor | C_14_H_10_F_3_NO_2_ | 281 |  | Williams et al. (2015) |
| 75 |  | Succinic acid, 3,5-dinitrobenzyl 2-methylhex-3-yl ester | Enzyme activator | C_18_H_24_N_2_O_8_ | 396 |  | Martinez et al. (2008) |
| 76 |  | Oxalic acid, monoamide, N-(2-fluorophenyl)-, heptyl ester | Antioxidant | C_15_H_20_FNO_3_ | 281 | 1.5 | Ganyam et al. (2019) |
| 77 |  | Propanamide, 2,2,3,3,3-pentafluoro-N-(2,4,6-trimethylphenyl)- | Inhibitor | C_12_H_12_F_5_NO | 281 |  | Talley et al. (2000) |
| 78 | 12.3 | 3-Trifluoroacetoxypentadecane | Antimicrobial | C_17_H_31_F_3_O_2_ | 324 | 0.7 | Hussein et al. (2016) |
| 79 |  | Z-10-Tetradecen-1-ol acetate | Pharmaceutical | C_16_H_30_O_2_ | 254 |  | Hameed et al. (2015) |
| 80 |  | Dodecanoic acid, 3-hydroxy- | Cytotoxic | C_12_H_24_O_3_ | 216 |  | Viegas et al. (1989) |
| 81 |  | 3-Trifluoroacetoxydodecane | Antioxidant | C_14_H_25_F_3_O_2_ | 282 |  | Zagulyaeva et al. (2010) |
| 82 |  | 10-Undecenoic acid, octyl ester | Antimicrobial | C_19_H_36_O_2_ | 296 |  | Van der Steen and Stevens (2009) |
| 83 |  | 3-Cyclopropylcarbonyloxydodecane | Reducing Agent | C_16_H_30_O_2_ | 254 |  | Bolade et al. (2018) |
| 84 | 13.3 | 1-Hexadecanol, 2-methyl- | Antioxidant | C_17_H_36_O | 256 | 0.2 | Hussein et al. (2015) |
| 85 |  | 1-Dodecanol, 3,7,11-trimethyl- | Cytotoxic | C_15_H_32_O | 228 |  | Fahem et al. (2020) |
| 86 |  | Pentadecanoic acid | Oxidation | C_15_H_30_O_2_ | 242 |  | Jenkins et al. (2015) |
| 87 |  | Hexadecane, 1,1-bis(dodecyloxy)- | Antioxidant | C_40_H_82_O_2_ | 594 |  | Ser et al. (2015) |
| 88 |  | Cyclopropanetetradecanoic acid, 2-octyl-, methyl ester | Pharmacological | C_26_H_50_O_2_ | 394 |  | Srivastava et al. (2015) |
| 89 |  | Heptadecanoic acid, heptadecyl ester | Antimicrobial | C_34_H_68_O_2_ | 508 |  | Gautam et al. (2016) |
| 90 |  | 2-Myristynoyl pantetheine |  | C_25_H_44_N_2_O_5_S | 484 |  | Srivastava et al. (2015) |
| 91 | 13.6 | Oleic Acid | Anti-tumor | C_18_H_34_O_2_ | 282 | 1.9 | Carrillo Pérez et al. (2012) |
| 92 |  | 13-Octadecenoic acid | Anti-protozoal | C_18_H_34_O_2_ | 282 |  | Carballeira et al. (2009) |
| 93 |  | cis-Vaccenic acid | Protects from Heart failure | C_18_H_34_O_2_ | 282 |  | Djoussé et al. (2014) |
| 94 |  | 9-Octadecenoic acid, (E)- | Inhibitor | C_18_H_34_O_2_ | 282 |  | Carballeira et al. (2009) |
| 95 |  | 9-Hexadecenoic acid | Cosmetics | C_16_H_30_O_2_ | 254 |  | Takigawa et al. (2005) |
| 96 |  | cis-13-Eicosenoic acid | Anti-obesity | C_20_H_38_O_2_ | 310 |  | Senarath et al. (2018) |
| 97 |  | 3-Heptafluorobutyroxytetradecane | Polymerization | C_18_H_29_F_7_O_2_ | 410 |  | MacKenzie and Tenaschuk (1979) |
| 98 |  | n-Nonadecanol-1 | Antifeedant | C_19_H_40_O | 284 |  | Aznar-Fernández et al. (2019) |

**References**

Abdillah, S., Tambunan, R. M., Farida, Y., Sandhiutami, N. M. D., and Dewi, R. M. (2015). Phytochemical screening and antimalarial activity of some plants traditionally used in Indonesia. *Asian Pacific Journal of Tropical Disease* 5, 454–457.

Abou-Ghadir, O., M Hayallah, A., Abdel-Moty, S., and Hussein, M. (2014). Design and synthesis of some new purine-dione derivatives of potential anti-inflammatory activity. *Der Pharma Chemica* 6, 199–211.

Achimón, F., Brito, V. D., Pizzolitto, R. P., and Zygadlo, J. A. (2022). Effect of Carbon Sources on the Production of Volatile Organic Compounds by Fusarium verticillioides. *Journal of Fungi* 8, 158. doi: 10.3390/jof8020158.

Akocak, S., Taslimi, P., Lolak, N., Işık, M., Durgun, M., Budak, Y., et al. (2021). Synthesis, Characterization, and Inhibition Study of Novel Substituted Phenylureido Sulfaguanidine Derivatives as α-Glycosidase and Cholinesterase Inhibitors. *Chemistry & Biodiversity* 18, e2000958. doi: 10.1002/cbdv.202000958.

Alam, F., Din, K., Rasheed, R., Sadiq, A., Jan, M., Minhas, A. M., et al. (2020). Phytochemical investigation, anti-inflammatory, antipyretic and antinociceptive activities of Zanthoxylum armatum DC extracts-in vivo and in vitro experiments. *Heliyon* 6, e05571. doi: 10.1016/j.heliyon.2020.e05571.

Al-Taisan, K. M., Al-Hazimi, H. M. A., and Al-Shihry, S. S. (2010). Synthesis, Characterization and Biological Studies of Some Novel Thieno[2,3-d]pyrimidines. *Molecules* 15, 3932–3957. doi: 10.3390/molecules15063932.

Alves-Santos, T. R., Silva, O. A., Moreira, H. S., Borges, R. S., Duarte, G. P., Magalhães, P. J. C., et al. (2019). Cardiovascular Effects of Trans-4-Methoxy-β-Nitrostyrene in Spontaneously Hypertensive Rats: Comparison with Its Parent Drug β-Nitrostyrene. *Front Pharmacol* 10, 1407. doi: 10.3389/fphar.2019.01407.

Anand, S. S., Philip, B. K., and Mehendale, H. M. (2014). “Chlorination Byproducts,” in *Encyclopedia of Toxicology (Third Edition)*, ed. P. Wexler (Oxford: Academic Press), 855–859. doi: 10.1016/B978-0-12-386454-3.00276-1.

Anderson, C. E., Donde, Y., Douglas, C. J., and Overman, L. E. (2005). Catalytic Asymmetric Synthesis of Chiral Allylic Amines. Evaluation of Ferrocenyloxazoline Palladacycle Catalysts and Imidate Motifs. *J. Org. Chem.* 70, 648–657. doi: 10.1021/jo048490r.

Arulmurugan, S., and Kavitha, H. (2010). 2-Methyl-3-{4-[2-(1H-tetrazol-5-yl)ethylamino]phenyl}-3H-quinazolin-4-one. *Molbank* 2010. doi: 10.3390/M695.

Assassi, N., TAZEROUTI, A., and Canselier, J. (2005). Analysis of chlorinated, sulfochlorinated and sulfonamide derivatives of n-tetradecane by gas chromatography/mass spectrometry. *Journal of chromatography. A* 1071, 71–80. doi: 10.1016/j.chroma.2005.01.102.

Aznar-Fernández, T., Cimmino, A., Masi, M., Rubiales, D., and Evidente, A. (2019). Antifeedant activity of long-chain alcohols, and fungal and plant metabolites against pea aphid (Acyrthosiphon pisum) as potential biocontrol strategy. *Natural Product Research* 33, 2471–2479. doi: 10.1080/14786419.2018.1452013.

Bolade, O. P., Akinsiku, A. A., Adeyemi, A. O., Williams, A. B., and Benson, N. U. (2018). Dataset on phytochemical screening, FTIR and GC–MS characterisation of Azadirachta indica and Cymbopogon citratus as reducing and stabilising agents for nanoparticles synthesis. *Data in Brief* 20, 917–926. doi: 10.1016/j.dib.2018.08.133.

Campos, J. F., Besson, T., and Berteina-Raboin, S. (2022). Review on the Synthesis and Therapeutic Potential of Pyrido[2,3-d], [3,2-d], [3,4-d] and [4,3-d]pyrimidine Derivatives. *Pharmaceuticals* 15, 352. doi: 10.3390/ph15030352.

Carballeira, N. M., Montano, N., Balaña-Fouce, R., and Prada, C. F. (2009). First total synthesis and antiprotozoal activity of (Z)-17-methyl-13-octadecenoic acid, a new marine fatty acid from the sponge Polymastia penicillus. *Chemistry and Physics of Lipids* 161, 38–43. doi: 10.1016/j.chemphyslip.2009.06.140.

Carrillo Pérez, C., Cavia Camarero, M. del M., and Alonso de la Torre, S. (2012). Antitumor effect of oleic acid; mechanisms of action. A review. *Efecto antitumoral del ácido oleico; mecanismos de acción: revisión científica*. doi: 10.3305/nh.2012.27.6.6010.

Chagnes, A., Rager, M.-N., Courtaud, B., Thiry, J., and Cote, G. (2010). Speciation of vanadium (V) extracted from acidic sulfate media by trioctylamine in n-dodecane modified with 1-tridecanol. *Hydrometallurgy* 104, 20–24. doi: 10.1016/j.hydromet.2010.04.004.

Cullere, L., Cacho, J., and Ferreira, V. (2004). Analysis for wine C5–C8 aldehydes through the determination of their O-(2,3,4,5,6-pentafluorobenzyl)oximes formed directly in the solid phase extraction cartridge. *Analytica Chimica Acta* 524, 201–206. doi: 10.1016/j.aca.2004.03.025.

Damiano, F., De Benedetto, G. E., Longo, S., Giannotti, L., Fico, D., Siculella, L., et al. (2020). Decanoic Acid and Not Octanoic Acid Stimulates Fatty Acid Synthesis in U87MG Glioblastoma Cells: A Metabolomics Study. *Frontiers in Neuroscience* 14. Available at: https://www.frontiersin.org/articles/10.3389/fnins.2020.00783 [Accessed September 3, 2022].

Daniels, A., and Temikotan, T. (2021). Fatty acid profile, antioxidant and antibacterial effect of the ethyl acatate extract of cleistopholis patens. *Bulletin of Scientific Research* 3, 21–31. doi: 10.34256/bsr2113.

de Bony, J., Martin, G., Welby, M., and Tocanne, J. F. (1984). Evidence for a homogeneous lateral distribution of lipids in a bacterial membrane: A photo cross-linking approach using anthracene as a photoactivable group. *FEBS Letters* 174, 1–6. doi: 10.1016/0014-5793(84)81065-0.

Djoussé, L., Matsumoto, C., Hanson, N. Q., Weir, N. L., Tsai, M. Y., and Gaziano, J. M. (2014). Plasma cis-vaccenic acid and risk of heart failure with antecedent coronary heart disease in male physicians. *Clinical Nutrition* 33, 478–482. doi: 10.1016/j.clnu.2013.07.001.

EL-Hashash, M. A., Essawy, A., and Sobhy Fawzy, A. (2014). Synthesis and Antimicrobial Activity of Some Novel Heterocyclic Candidates via Michael Addition Involving 4-(4-Acetamidophenyl)-4-oxobut-2-enoic Acid. *Advances in Chemistry* 2014, e619749. doi: 10.1155/2014/619749.

Ezekwe, S., Rizwan, A., Rabiu, K., and Ogbonnaya, E. (2020). Qualitative phytochemical and GC-MS analysis of some commonly consumed vegetables. *GSC Biological and Pharmaceutical Sciences* 12, 208–214. doi: 10.30574/gscbps.2020.12.3.0299.

Fahem, N., Djellouli, A. S., and Bahri, S. (2020). Cytotoxic Activity Assessment and GC-MS Screening of Two Codium Species Extracts. *Pharmaceutical Chemistry Journal* 54, 755–760. doi: 10.1007/s11094-020-02266-z.

Fang, M.-Y., Chen, L.-P., Huang, L., Fang, D.-M., Chen, X.-Z., Wang, B.-Q., et al. (2021). Synthesis of Tribenzo[b,d,f]azepines via Palladium-Catalyzed Annulation Reaction of 2-Iodobiphenyls with 2-Halogenoanilines. *J. Org. Chem.* 86, 9096–9106. doi: 10.1021/acs.joc.1c01082.

Francke, W., and Schulz, S. (1999). “8.04 - Pheromones,” in *Comprehensive Natural Products Chemistry*, eds. S. D. Barton, K. Nakanishi, and O. Meth-Cohn (Oxford: Pergamon), 197–261. doi: 10.1016/B978-0-08-091283-7.00052-7.

Fuentes, A. S., del Pozo Losada, C., and Vora, H. U. (2016). “(−)-(4R,5R)-4,5-Bis[hydroxy(diphenyl)methyl]-2,2-dimethyl-1,3-dioxolane,” in *Encyclopedia of Reagents for Organic Synthesis* (John Wiley & Sons, Ltd), 1–9. doi: 10.1002/047084289X.rn00861.pub2.

Gad, S. E. (2005). “Hydroxylamine,” in *Encyclopedia of Toxicology (Second Edition)*, ed. P. Wexler (New York: Elsevier), 557–558. doi: 10.1016/B0-12-369400-0/00508-1.

Gad, S. E. (2014). “Hydroperoxide, tert-Butyl,” in *Encyclopedia of Toxicology (Third Edition)*, ed. P. Wexler (Oxford: Academic Press), 977–978. doi: 10.1016/B978-0-12-386454-3.00854-X.

Gadhi, A., El-Sherbiny, M., Al-Sofynai, A., Baakdah, M., and Sathianeson, S. (2019). Antimicrofouling activities of marine macroalga Dictyota dichotoma from the Red Sea. *Journal of Agricultural and Marine Sciences [JAMS]* 23, 58. doi: 10.24200/jams.vol23iss1pp58-67.

Ganesh, M., and Mohankumar, M. (2017). Extraction and identification of bioactive components in Sida cordata (Burm.f.) using gas chromatography–mass spectrometry. *J Food Sci Technol* 54, 3082–3091. doi: 10.1007/s13197-017-2744-z.

Ganyam, M., Anaduaka, E., Gabriel, F., Sani, S., and Fedilis, I. (2019). Effects of Methanol Extract of Toasted African Yam Bean Seeds (Sphenostylis stenocarpa) on Anti-Inflammatory Properties.

Gautam, V., Sharma, A., Arora, S., and Bhardwaj, R. (2016). Bioactive compounds in the different extracts of flowers of Rhododendron arboreum Sm. *Journal of Chemical and Pharmaceutical Research* 2016, 439–444.

Gensler, W. J., and Thomas, G. R. (1952). Synthesis of Unsaturated Fatty Acids: Vaccenic Acid. *Journal of the American Chemical Society* 74, 3942–3943.

Gil, S., Lázaro, M. A., Mestres, R., Millan, F., and Parra, M. (1995). Components of the Sex Pheromone of Chilo Supressalis: Efficient Syntheses of (Z)-11-Hexadecenal and (Z)-13-Octadecenal. *Synthetic Communications* 25, 351–361. doi: 10.1080/00397919508011366.

Godwin, J., Chukwu, U. J., and Gad, T. (2012). Distribution of Iron (II) Between Buffered Aqueous Solutions and Chloroform Solution of N, N’-Ethylenebis (4-Butanoyl-2,4-Dihydro-5-Methyl-2-Phenyl-3h-Pyrazol-3-Oneimine). *JOURNAL OF ADVANCES IN CHEMISTRY* 8, 1581–1589. doi: 10.24297/jac.v8i2.4039.

Gülerman, N., Oruç-Emre, E., Kartal, F., and Rollas, S. (2000). In vivo metabolism of 4-fluorobenzoic acid [(5-nitro-2-furanyl)methylene] hydrazide in rats. *European journal of drug metabolism and pharmacokinetics* 25, 103–8. doi: 10.1007/BF03190075.

Halling, P. J., Ross, A. C., and Bell, G. (1998). “Inactivation of enzymes at the aqueous-organic interface,” in *Progress in Biotechnology* Stability and Stabilization of Biocatalysts., eds. A. Ballesteros, F. J. Plou, J. L. Iborra, and P. J. Halling (Elsevier), 365–372. doi: 10.1016/S0921-0423(98)80054-3.

Ham, J. E., and Raymond Wells, J. (2009). Surface chemistry of dihydromyrcenol (2,6-dimethyl-7-octen-2-ol) with ozone on silanized glass, glass, and vinyl flooring tiles. *Atmospheric Environment* 43, 4023–4032. doi: 10.1016/j.atmosenv.2009.05.007.

Hameed, I., J. Hussein, H., Kareem, M., and Hamad, N. (2015). Identification of five newly described bioactive chemical compounds in Methanolic extract of Mentha viridis by using gas chromatography – mass spectrometry (GC-MS). *Journal of Pharmacognosy and Phytotherapy* 7, 107–125. doi: 10.5897/JPP2015.0349.

Hasan Tanak, Karataş, Ş., Meral, S., and Ağar, A. A. (2020). Synthesis, Molecular Structure and Quantum Chemical Studies of N-(2-Fluorophenyl)-1-(5-Nitrothiophen-2-yl)methanimine. *Crystallogr. Rep.* 65, 1212–1216. doi: 10.1134/S106377452007024X.

Hušek, P., Švagera, Z., Hanzlíková, D., Řimnáčová, L., Zahradníčková, H., Opekarová, I., et al. (2016). Profiling of urinary amino-carboxylic metabolites by in-situ heptafluorobutyl chloroformate mediated sample preparation and gas chromatography–mass spectrometry. *Journal of Chromatography A* 1443, 211–232. doi: 10.1016/j.chroma.2016.03.019.

Hussein, A. O., Hameed*, I. H., Jasim, H., and Kareem, M. A. (2015). Determination of alkaloid compounds of Ricinus communis by using gas chromatography- mass spectroscopy (GC-MS). *JMPR* 9, 349–359. doi: 10.5897/JMPR2015.5750.

Hussein, H., Hameed, I., and Ibraheem, O. A. (2016). Antimicrobial activity and spectral chemical analysis of methanolic leaves extract of adiantum capillus-veneris using GC-MS and FT-IR spectroscopy. 8, 369–385.

Idan, S., Al-Marzoqi, A., and Hameed, I. (2015). Spectral analysis and anti-bacterial activity of methanolic fruit extract of Citrullus colocynthis using gas chromatography-mass spectrometry. *African Journal of Biotechnology* 14, 3131–3158. doi: 10.5897/AJB2015.14957.

Jachak, M., Bhusnar, A., Medhane, V., and Toche, R. (2006). A Convenient Route for the Synthesis of Pyrazolo[3,4-d]pyrimidine, Pyrazolo[3,4-b][1,6]naphthyridine and Pyrazolo[3,4-b]quinoline Derivatives. *Journal of Heterocyclic Chemistry* 43, 1169–1175. doi: 10.1002/jhet.5570430506.

Jahan, I., Tona, M. R., Sharmin, S., Sayeed, M. A., Tania, F. Z., Paul, A., et al. (2020). GC-MS Phytochemical Profiling, Pharmacological Properties, and In Silico Studies of Chukrasia velutina Leaves: A Novel Source for Bioactive Agents. *Molecules* 25, 3536. doi: 10.3390/molecules25153536.

Jamieson, S. M. F., Brooke, D. G., Heinrich, D., Atwell, G. J., Silva, S., Hamilton, E. J., et al. (2012). 3-(3,4-Dihydroisoquinolin-2(1H)-ylsulfonyl)benzoic Acids: Highly Potent and Selective Inhibitors of the Type 5 17-β-Hydroxysteroid Dehydrogenase AKR1C3. *J. Med. Chem.* 55, 7746–7758. doi: 10.1021/jm3007867.

Jenkins, B., West, J. A., and Koulman, A. (2015). A Review of Odd-Chain Fatty Acid Metabolism and the Role of Pentadecanoic Acid (C15:0) and Heptadecanoic Acid (C17:0) in Health and Disease. *Molecules* 20, 2425–2444. doi: 10.3390/molecules20022425.

Jubete, G., Puig de la Bellacasa, R., Estrada-Tejedor, R., Teixidó, J., and Borrell, J. I. (2019). Pyrido[2,3-d]pyrimidin-7(8H)-ones: Synthesis and Biomedical Applications. *Molecules* 24, 4161. doi: 10.3390/molecules24224161.

K., S., Bhargavan, R., and Kasinathan, S. (2016). PHYTOCHEMICAL SCREENING AND GC-MS ANALYSIS OF ETHANOLIC EXTRACT OF TRIBULUS TERRESTRIS. *International Journal of Pharmacology Research* 6, 44–50.

Krátký, M., Konečná, K., Janoušek, J., Brablíková, M., Janďourek, O., Trejtnar, F., et al. (2019). 4-Aminobenzoic Acid Derivatives: Converting Folate Precursor to Antimicrobial and Cytotoxic Agents. *Biomolecules* 10, 9. doi: 10.3390/biom10010009.

Kroumova, A. B., and Wagner, G. J. (2003). Different elongation pathways in the biosynthesis of acyl groups of trichome exudate sugar esters from various solanaceous plants. *Planta* 216, 1013–1021. doi: 10.1007/s00425-002-0954-7.

Kula, J., Quang, T. B., and Smigielski, K. (2001). Convenient Synthesis of (r)-1,3-Nonanediol. *Synthetic Communications* 31, 463–467. doi: 10.1081/SCC-100000540.

Lemieux, R. U., and Nagabhushan, T. L. (1968). The synthesis of 2-amino-2-deoxyhexoses: D-glucosamine, D-mannosamine, D-galactosamine, and D-talosamine. *Can. J. Chem.* 46, 401–403. doi: 10.1139/v68-064.

Ley, S. V., and Madin, A. (1991). “2.7 - Oxidation Adjacent to Oxygen of Alcohols by Chromium Reagents,” in *Comprehensive Organic Synthesis*, eds. B. M. Trost and I. Fleming (Oxford: Pergamon), 251–289. doi: 10.1016/B978-0-08-052349-1.00190-6.

MacKenzie, S. L., and Tenaschuk, D. (1979). Quantitative fromation of N(O,S)-heptafluorobutyryl isobutyl amino acids for gas chromatographic analysis: I. Esterification. *Journal of Chromatography A* 171, 195–208. doi: 10.1016/S0021-9673(01)95299-9.

Martín-Acosta, P., Amesty, Á., Guerra-Rodríguez, M., Guerra, B., Fernández-Pérez, L., and Estévez-Braun, A. (2021). Modular Synthesis and Antiproliferative Activity of New Dihydro-1H-pyrazolo[1,3-b]pyridine Embelin Derivatives. *Pharmaceuticals (Basel)* 14, 1026. doi: 10.3390/ph14101026.

Martinez, C., Hu, S., Dumond, Y., Tao, J., Kelleher, P., and Tully, L. (2008). Development of a Chemoenzymatic Manufacturing Process for Pregabalin. *Organic Process Research & Development - ORG PROCESS RES DEV* 12. doi: 10.1021/op7002248.

Millero, F. J., Graham, T. B., Huang, F., Bustos-Serrano, H., and Pierrot, D. (2006). Dissociation constants of carbonic acid in seawater as a function of salinity and temperature. *Marine Chemistry* 100, 80–94. doi: 10.1016/j.marchem.2005.12.001.

Moldoveanu, S. C. (2019). “Chapter 2 - Pyrolysis of Hydrocarbons,” in *Pyrolysis of Organic Molecules (Second Edition)*, ed. S. C. Moldoveanu (Elsevier), 35–161. doi: 10.1016/B978-0-444-64000-0.00002-0.

Ozden, S., Oztürk, A., Goker, H., and Altanlar, N. (2000). Synthesis and antimicrobial activity of some new 4-hydroxy-2H-1,4-benzoxazin-3(4H)-ones. *Farmaco (Società chimica italiana : 1989)* 55, 715–8. doi: 10.1016/S0014-827X(00)00098-7.

Palanki, M., Erdman, P. E., Goldman, M. E., Suto, C., and Suto, M. J. (2000). Synthesis and structure-activity relationship studies of conformationally restricted, analogs of 2-chloro-4-trifluoromethylpyrimidine- 5-[N-(3’,5’-bis(trifluoromethyl)phenyl)]carboxamide. *Medicinal Chemistry Research* 10, 19–29.

Patel, A. V., Malik, G., and Bhatt, P. (2009). Synthesis and characterization of some novel Biphenyl 4-carboxylic acid((3-chloro 2-(substituted phenyl) - 4-oxo azetidine-1-yl) amide. *Oriental Journal of Chemistry* 25, 775–778.

Qiu, H., Liu, R., and Long, L. (2019). Analysis of Chemical Composition of Extractives by Acetone and the Chromatic Aberration of Teak (Tectona Grandis L.F.) from China. *Molecules* 24, 1989. doi: 10.3390/molecules24101989.

Rebbert, R. E., and Ausloos, P. (1962). Intramolecular Rearrangements in the Solid Phase Photolysis of 4‐Methyl‐2‐Hexanone and sec‐Butyl Acetate. *J. Chem. Phys.* 37, 1158–1159. doi: 10.1063/1.1733239.

Reddy, N., and Ananthaprasad, M. G. (2021). “Chapter 11 - Polymeric materials for three-dimensional printing,” in *Additive Manufacturing* Woodhead Publishing Reviews: Mechanical Engineering Series., eds. M. Manjaiah, K. Raghavendra, N. Balashanmugam, and J. P. Davim (Woodhead Publishing), 233–274. doi: 10.1016/B978-0-12-822056-6.00010-2.

Rossi, A., Martins, M. P., Bitencourt, T. A., Peres, N. T. A., Rocha, C. H. L., Rocha, F. M. G., et al. (2021). Reassessing the Use of Undecanoic Acid as a Therapeutic Strategy for Treating Fungal Infections. *Mycopathologia* 186, 327–340. doi: 10.1007/s11046-021-00550-4.

Schwabe, A. D., Bennett, L. R., and Bowman, L. P. (1964). Octanoic acid absorption and oxidation in humans. *Journal of Applied Physiology* 19, 335–337. doi: 10.1152/jappl.1964.19.2.335.

Senarath, S., Yoshinaga, K., Nagai, T., Yoshida, A., Beppu, F., and Gotoh, N. (2018). Differential Effect of cis-Eicosenoic Acid Positional Isomers on Adipogenesis and Lipid Accumulation in 3T3-L1 Cells. *European Journal of Lipid Science and Technology* 120, 1700512. doi: 10.1002/ejlt.201700512.

Ser, H. L., Palanisamy, U., Yin, W.-F., Abd Malek, N., Chan, K.-G., Goh, B. H., et al. (2015). Presence of antioxidative agent, Pyrrolo[1,2-a]pyrazine-1,4-dione, hexahydro- in newly isolated Streptomyces mangrovisoli sp. nov. *Frontiers in microbiology* 6, 854. doi: 10.3389/fmicb.2015.00854.

Shalini, K., Sharma, P. K., and Kumar, N. (2010). Imidazole and its biological activities: A review. 13.

Simon, A., and Losada, C. (2008). “(−)-(4 R ,5 R )-4,5-Bis[hydroxy(diphenyl)methyl]-2,2-dimethyl-1,3-dioxolane,” in doi: 10.1002/047084289X.rn00861.

Sivaprakash, S., Prakash, S., Mohan, S., and Jose, S. P. (2019). Quantum chemical studies and spectroscopic investigations on 2-amino-3-methyl-5-nitropyridine by density functional theory. *Heliyon* 5, e02149. doi: 10.1016/j.heliyon.2019.e02149.

Skhirtladze, L., Lietonas, K., Bucinskas, A., Volyniuk, D., Mahmoudi, M., Mukbaniani, O., et al. (2022). 1,4-Bis(trifluoromethyl)benzene as a new acceptor for the design and synthesis of emitters exhibiting efficient thermally activated delayed fluorescence and electroluminescence: experimental and computational guidance. *J. Mater. Chem. C* 10, 4929–4940. doi: 10.1039/D1TC05420A.

Srivastava, R., Mukerjee, A., and Verma, A. (2015a). GC-MS Analysis of Phytocomponents in, Pet Ether Fraction of Wrightia tinctoria Seed. *Pharmacognosy Journal* 7, 249–253. doi: 10.5530/pj.2015.4.7.

Srivastava, R., Mukerjee, A., and Verma, A. (2015b). GC-MS Analysis of Phytocomponents in, Pet Ether Fraction of Wrightia tinctoria Seed. *Pharmacognosy Journal* 7, 249–253. doi: 10.5530/pj.2015.4.7.

Su, X., Halem, H. A., Thomas, M. P., Moutrille, C., Culler, M. D., Vicker, N., et al. (2012). Adamantyl carboxamides and acetamides as potent human 11β-hydroxysteroid dehydrogenase type 1 inhibitors. *Bioorg Med Chem* 20, 6394–6402. doi: 10.1016/j.bmc.2012.08.056.

Takigawa, H., Nakagawa, H., Kuzukawa, M., Mori, H., and Imokawa, G. (2005). Deficient Production of Hexadecenoic Acid in the Skin Is Associated in Part with the Vulnerability of Atopic Dermatitis Patients to Colonization by Staphylococcus aureus. *DRM* 211, 240–248. doi: 10.1159/000087018.

Talbaoui, A., Hamdaoui, L., Bouyahya, A., El Moussaouiti, M., and Bakri, Y. (2020). Chemical Composition, in vitro Cytotoxic, and Antibacterial Activities of Moroccan Medicinal Plants Euphorbia resinifera and Marrubium vulgare. *Biointerface Research in Applied Chemistry* 10, 7343–7355. doi: 10.33263/BRIAC106.73437355.

Talley, J. J., Bertenshaw, S. R., Brown, D. L., Carter, J. S., Graneto, M. J., Kellogg, M. S., et al. (2000). N-[[(5-Methyl-3-phenylisoxazol-4-yl)- phenyl]sulfonyl]propanamide, Sodium Salt, Parecoxib Sodium:  A Potent and Selective Inhibitor of COX-2 for Parenteral Administration. *J. Med. Chem.* 43, 1661–1663. doi: 10.1021/jm000069h.

Tanaka, T., and Hayashi, M. (2008). Catalytic Enantioselective Reformatsky Reaction of Alkyl Iodoacetate with Aldehydes Catalyzed by Chiral Schiff Base. *Chem. Lett.* 37, 1298–1299. doi: 10.1246/cl.2008.1298.

Tsunoda, H., Kudo, T., Masaki, Y., Ohkubo, A., Seio, K., and Sekine, M. (2011). Biochemical behavior of N-oxidized cytosine and adenine bases in DNA polymerase-mediated primer extension reactions. *Nucleic Acids Res* 39, 2995–3004. doi: 10.1093/nar/gkq914.

Van der Steen, M., and Stevens, C. V. (2009). Undecylenic Acid: A Valuable and Physiologically Active Renewable Building Block from Castor Oil. *ChemSusChem* 2, 692–713. doi: 10.1002/cssc.200900075.

Viegas, C. A., Rosa, M. F., Sá-Correia, I., and Novais, J. M. (1989). Inhibition of Yeast Growth by Octanoic and Decanoic Acids Produced during Ethanolic Fermentation. *Applied and Environmental Microbiology* 55, 21–28. doi: 10.1128/aem.55.1.21-28.1989.

Wang, Y., Zhang, L.-T., Feng, Y.-X., Guo, S.-S., Pang, X., Zhang, D., et al. (2019). Insecticidal and repellent efficacy against stored-product insects of oxygenated monoterpenes and 2-dodecanone of the essential oil from Zanthoxylum planispinum var. dintanensis. *Environ Sci Pollut Res* 26, 24988–24997. doi: 10.1007/s11356-019-05765-z.

Williams, J. D., Torhan, M. C., Neelagiri, V., Brown, C., Bowlin, N. O., Di, M., et al. (2015). Synthesis and structure-activity relationships of novel phenoxyacetamide inhibitors of the Pseudomonas aeruginosa type III secretion system (T3SS). *Bioorg Med Chem* 23, 1027–1043. doi: 10.1016/j.bmc.2015.01.011.

Xie, F.-M., Li, H.-Z., Dai, G.-L., Li, Y.-Q., Cheng, T., Xie, M., et al. (2019). Rational Molecular Design of Dibenzo[a,c]phenazine-Based Thermally Activated Delayed Fluorescence Emitters for Orange-Red OLEDs with EQE up to 22.0%. *ACS Appl. Mater. Interfaces* 11, 26144–26151. doi: 10.1021/acsami.9b06401.

Xue, J., Zhuo, J., Liu, M., Chi, Y., Zhang, D., and Yao, Q. (2017). Synergetic Effect of Co-pyrolysis of Cellulose and Polypropylene over an All-Silica Mesoporous Catalyst MCM-41 Using Thermogravimetry–Fourier Transform Infrared Spectroscopy and Pyrolysis–Gas Chromatography–Mass Spectrometry. *Energy Fuels* 31, 9576–9584. doi: 10.1021/acs.energyfuels.7b01651.

Zagulyaeva, A. A., Yusubov, M. S., and Zhdankin, V. V. (2010). A General and Convenient Preparation of [Bis(trifluoroacetoxy)iodo]perfluoroalkanes and [Bis(trifluoroacetoxy)iodo]arenes by Oxidation of Organic Iodides Using Oxone and Trifluoroacetic Acid. *J. Org. Chem.* 75, 2119–2122. doi: 10.1021/jo902733f.

Zeitoun, M., Adel, M., Abulfotouh, F., and Ebrahim, S. (2021). Thermophysical properties enhancement of octadecane using reduced graphene oxide and graphene oxide nanoplatelets. *Journal of Energy Storage* 38, 102512. doi: 10.1016/j.est.2021.102512.

Zhang, L., Peng, X.-M., Damu, G. L. V., Geng, R.-X., and Zhou, C.-H. (2014). Comprehensive Review in Current Developments of Imidazole-Based Medicinal Chemistry. *Medicinal Research Reviews* 34, 340–437. doi: 10.1002/med.21290.

Zhang, X., Wang, Y.-X., Zhao, J., Duan, P., Chen, Y., and Chen, L. (2017). Structural Insights Into 9-Styrylanthracene-Based Luminophores: Geometry Control Versus Mechanofluorochromism and Sensing Properties. *Chemistry – An Asian Journal* 12, 830–834. doi: 10.1002/asia.201700183.

Zibuck, R. (2001). “2-Acetoxyisobutyryl Chloride,” in *Encyclopedia of Reagents for Organic Synthesis* (John Wiley & Sons, Ltd). doi: 10.1002/047084289X.ra022.
